# Autofluorescence imaging of endogenous metabolic cofactors in response to cytokine stimulation of classically activated macrophages

**DOI:** 10.1186/s40170-023-00325-z

**Published:** 2023-11-13

**Authors:** Shelby N. Bess, Matthew J. Igoe, Abby C. Denison, Timothy J. Muldoon

**Affiliations:** Department of Biomedical Engineering, University of Arkansas, Fayetteville, AR USA

**Keywords:** Macrophage, NADH, Autofluorescence imaging, Metabolism, Lactate, Succinate, Seahorse extracellular flux

## Abstract

**Background:**

Macrophages are one of the most prevalent subsets of immune cells within the tumor microenvironment and perform a range of functions depending on the cytokines and chemokines released by surrounding cells and tissues. Recent research has revealed that macrophages can exhibit a spectrum of phenotypes, making them highly plastic due to their ability to alter their physiology in response to environmental cues. Recent advances in examining heterogeneous macrophage populations include optical metabolic imaging, such as fluorescence lifetime imaging (FLIM), and multiphoton microscopy. However, the method of detection for these systems is reliant upon the coenzymes NAD(P)H and FAD, which can be affected by factors other than cytoplasmic metabolic changes. In this study, we seek to validate these optical measures of metabolism by comparing optical results to more standard methods of evaluating cellular metabolism, such as extracellular flux assays and the presence of metabolic intermediates.

**Methods:**

Here, we used autofluorescence imaging of endogenous metabolic co-factors via multiphoton microscopy and FLIM in conjunction with oxygen consumption rate and extracellular acidification rate through Seahorse extracellular flux assays to detect changes in cellular metabolism in quiescent and classically activated macrophages in response to cytokine stimulation.

**Results:**

Based on our Seahorse XFP flux analysis, M0 and M1 macrophages exhibit comparable trends in oxygen consumption rate (OCR) and extracellular acidification rate (ECAR). Autofluorescence imaging of M0 and M1 macrophages was not only able to show acute changes in the optical redox ratio from pre-differentiation (0 hours) to 72 hours post-cytokine differentiation (M0: 0.320 to 0.258 and M1: 0.316 to 0.386), mean NADH lifetime (M0: 1.272 ns to 1.379 ns and M1: 1.265 ns to 1.206 ns), and A1/A2 ratio (M0: 3.452 to ~ 4 and M1: 3.537 to 4.529) but could also detect heterogeneity within each macrophage population.

**Conclusions:**

Overall, the findings of this study suggest that autofluorescence metabolic imaging could be a reliable technique for longitudinal tracking of immune cell metabolism during activation post-cytokine stimulation.

**Supplementary Information:**

The online version contains supplementary material available at 10.1186/s40170-023-00325-z.

## Background

Macrophages were first identified as immune cells within the innate immune system that can kill and phagocytose cancer cells, which promote antitumor functions such as metastasis, invasion, angiogenesis, and immunosuppression [1]. However, subsequent studies have shown that macrophages can promote pro-tumor functions [2]. More detailed studies have explored the biological complexity of how the tumor microenvironment can convert antitumor phenotype macrophages into pro-tumor macrophages, leading to a decrease in patient survival across many cancer types [3]. With the association between poor survival and high macrophage infiltration, these immune cells are emerging as promising targets for cancer therapy. For this reason, a greater understanding of the complex interactions between macrophages and their surrounding microenvironment is crucial to identifying how macrophages can be targeted for cancer therapy.

Recent studies have shown that macrophages rarely display one distinct phenotype, meaning that the complexity of these immune cells cannot undergo a binary classification [2, 4–7]. Macrophages are typically broadly classified into the M1 and M2 phenotypes, though more complex subclassifications exist. Bacterial products, such as lipopolysaccharides, and pro-inflammatory cytokines, such as interferon-𝛾 (IFN-𝛾) can induce the M1 phenotype. This phenotype can produce angiostatic factors associated with antitumor immunity, such as interleukin (IL)-12. On the other side of the spectrum, immunoregulatory cytokines can induce the M2 phenotype, such as IL-4 and IL-10. This phenotype is responsible for the secretion of pro-angiogenic (i.e., vascular endothelial growth factor (VEGF)) and tissue remodeling factors associated with pro-tumor function.

Recent studies have revealed that macrophages have distinct metabolic characteristics that can be correlated with their functional state. M1 macrophages typically express inducible nitric oxide synthase (iNOS) to produce nitric oxide (NO) and display enhanced glycolytic metabolism with an impaired tricarboxylic acid (TCA) cycle, leading to a decrease in oxidative phosphorylation (OXPHOS) [8, 9]. Glycolysis is a metabolic pathway that converts glucose to pyruvate to generate two molecules of adenosine triphosphate (ATP) per unit of glucose. Even though this process is inefficient, it provides metabolic intermediates to support the synthesis of ribose, amino acids, and fatty acids that are crucial for the metabolic reprogramming of the cell based on stimuli received by the microenvironment [10]. Generally, M1 macrophage metabolism changes from OXPHOS to glycolysis, which is accompanied by an increase in lactate release and a decrease in oxygen consumption rates. After entering the cell, glucose is converted to glucose-6-phosphate (G6P). The pentose phosphate pathway (PPP) branches after the first step of glycolysis. The PPP occurs in two phases. In the oxidative phase, energy from the conversion of glucose-6-phosphate into ribulose-5-phosphate is used to reduce nicotinamide adenine dinucleotide phosphate (NADP+) to NADPH. NADPH is then used by NADPH oxidase to generate reactive oxygen species (ROS) to kill pathogens and apoptotic cells [10, 11]. Glycolysis is a critical event for M1 macrophages, and the inhibition of glycolysis affects several M1 functions such as phagocytosis, ROS production, and the secretion of pro-inflammatory cytokines [12–14].

**Fig. 1:**
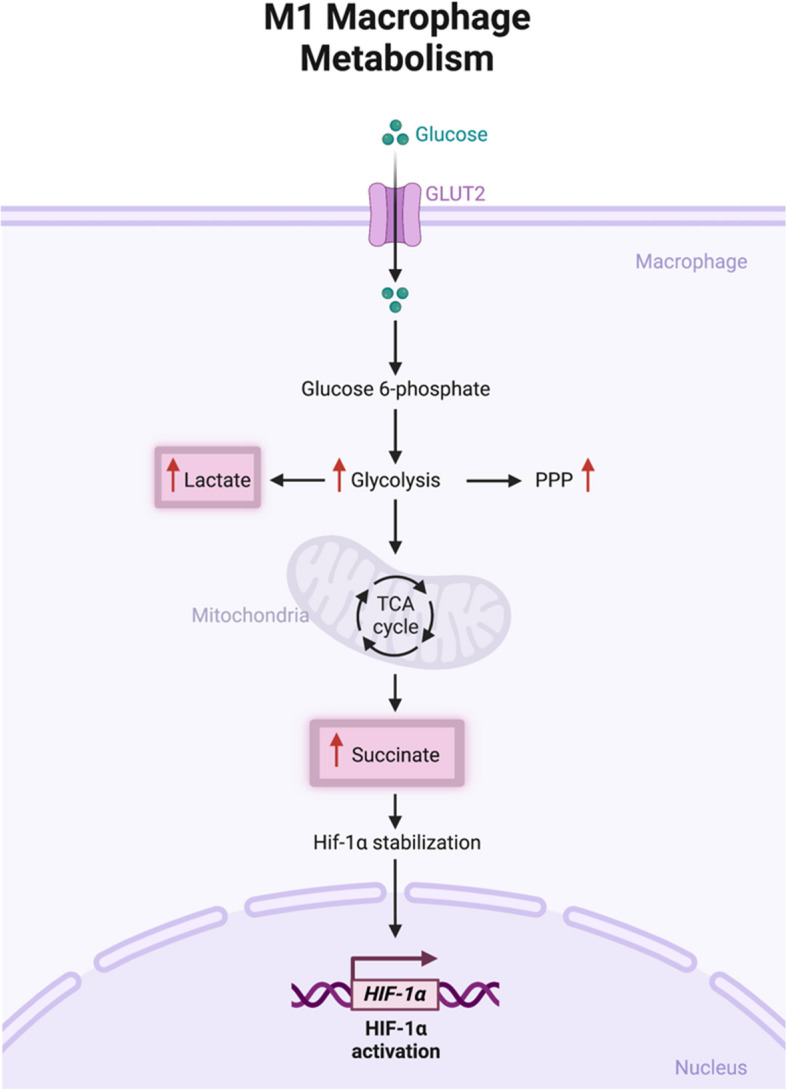
Schematic of M1 macrophage metabolism via glycolysis. Glucose is converted to glucose-6-phosphate through the PPP pathway, driving an increase in glycolysis. Maintenance of glycolytic metabolic reprogramming in M1 macrophages relies on the activation of several key transcription factors such as HIF-1𝛼, which facilitates rapid glucose uptake and supports the conversion of pyruvate from the TCA cycle to lactate. HIF-1𝛼 is stabilized by the accumulation of succinate from a break that occurs in the TCA cycle at succinate dehydrogenase. This leads to a release of ROS, leading to the activation of HIF-1𝛼. Figure was created with BioRender

Recent research has demonstrated that macrophages have unique metabolic characteristics that correlate with their functional status, termed as metabolic reprogramming [10]. Glycolytic metabolic reprogramming relies on the activation of several transcription factors, such as hypoxia-inducible factor 1-alpha (HIF-1α), which plays a key role in the commitment to glycolysis under normoxic conditions [10]. In M1 macrophages, HIF-1α acts as a metabolic and functional regulator of glycolytic genes such as glucose transporter-1 (GLUT-1), which facilitates rapid glucose uptake [10]. In addition, HIF-1α supports the conversion of pyruvate to lactate by promoting the expression of lactate dehydrogenase, limiting pyruvate from entering the TCA cycle [10]. Since OXPHOS is limited in M1 macrophages, this conversion is essential to restore NAD+ and maintain flux through the glycolytic pathway. HIF-1α can also be stabilized by the accumulation of succinate from the break at succinate dehydrogenase (SDH) in the TCA cycle [10]. Under hypoxic conditions, HIF-1𝛼 is stabilized against degradation, which transactivates and upregulates a series of genes that enable cells to adapt to reduced oxygen availability. During glycolysis, high amounts of succinate are oxidized to fumarate without ATP production, and electrons flux towards Complex I of the electron transport chain (ETC). This is associated with the release of ROS, leading to the activation of the transcription of HIF-1α. Succinate also works as a signaling molecule. Succinate influences HIF-1α stability by inhibiting prolyl hydroxylases (PHDs), a class of alpha-ketoglutarate (αKG)-dependent dioxygenases that regulate HIF-1α stability in an oxygen-dependent manner, blocking HIF-1α degradation in the presence of oxygen [10] (Fig. 1).

Assessment and quantification of cellular energy metabolism are essential for interrogating cellular functions [15–18]. Cells require energy in the form of ATP to support a variety of biological processes such as division, growth, differentiation, etc. [15, 16]. The study of cellular metabolism encompasses the biochemical pathways that generate and consume ATP as well as intermediate metabolites, carbon sources, signaling networks, and regulatory mechanisms that control these intertwined processes [15, 16]. For cells (and macrophages in particular) in culture, there are several methods that can be used to quantify either the metabolic intermediates present or changes in metabolic profiles over time. For example, M1 macrophages have been metabolically characterized as glycolytic if there is an accumulation of succinate based on the break in the TCA cycle at succinate dehydrogenase and the accumulation of lactate. Colorimetric or fluorometric analyzers can be used to quantify succinate and lactate levels within cell culture media [15]. Even though these measurements are relatively straightforward and nondestructive, they do not provide information on the possible fates of glucose-derived carbons or provide the ability to quantify succinate or lactate excretion at the single-cell level. Seahorse extracellular flux (XF) analyzers are also powerful tools to measure the oxygen consumption rate (OCR) and extracellular acidification rate (ECAR) of live cells, which are key indicators of mitochondrial respiration and glycolysis [15]. Optimization is often required when testing for the effect of cultured cells grown in the presence of compounds to ensure that these reagents do not interfere with the electron transport chain inhibitors and produce misleading data [15]. These include the trade-off between resolution and noise as well as the quantitative accuracy of the measurements [18]. Overall, these methods provide bulk readouts or averaging of phenotypes from individual cells within a sample [15]. Single-cell image analysis is an emerging approach for studying cell-to-cell heterogeneity [17], which can provide a more comprehensive understanding of key changes “cell-by-cell” rather than at the population level.

Optical imaging has emerged as a valuable tool in cellular and tissue biology due to its flexibility in revealing cellular biochemical details through light-matter interactions that include scattering, absorption, and fluorescence [19–21]. This allows for the longitudinal evaluation of biological processes [19]. A major advantage of optical imaging is the ability to resolve subcellular details with high-resolution microscopy techniques. These techniques can measure optical signals at specific wavelengths and, when combined with immunofluorescence labeling, can provide specificity in exploring cellular processes [17, 21]. In addition to the number of commercially available exogenous dyes and stains, cells can express several intrinsic fluorophores that play key roles in cellular metabolism and can be valuable in monitoring cellular metabolism [19]. Cellular redox networks responsible for metabolic control can adapt to changes in the cellular microenvironment to be able to maintain single-cell and multicellular processes responsible for growth and homeostasis [19, 22]. Pyridine nucleotides and flavoproteins have been identified as reliable intrinsic indicators of cellular metabolism. Nicotinamide adenine dinucleotide (NAD), a pyridine nucleotide that serves as a major metabolic electron carrier, can exist in reduced (NADH), oxidized (NAD+), and phosphorylated (NADPH) forms throughout the cell [19]. Similarly, the flavoprotein group flavin adenine dinucleotide (FAD) is also a metabolic electron carrier that can be in a reduced (FADH_2_) or oxidized (FAD) form. Out of these redox cofactors, NAD(P)H exhibits a higher fluorescence intensity within cells than FAD [19, 23]. The quantification of the fluorescence signals of these cofactors can provide an estimate of metabolic activity because metabolic potential coincides with the ratio of reduced and oxidized metabolic substrates. The use of high-resolution microscopy techniques enables an increased understanding of the heterogeneity of dynamic changes in redox states within cells and provides insight previously unachievable through other methods.

Advances in microscopy-based instrumentation now allow for deeper 3D visualization of cell redox states [19]. More specifically, multiphoton microscopy is particularly advantageous for NAD(P)H and FAD imaging. In multiphoton microscopy, molecules are brought to an excited state through the simultaneous absorption of two photons with half the energy and twice the wavelength. Therefore, near-infrared light can be used to excite FAD and NAD(P)H rather than high-energy ultraviolet (UV) and near-UV light [20]. The probability of near-simultaneous absorption of two photons is limited to the focal plane. This provides an intrinsic depth sectioning ability without the need for a confocal pinhole, allowing for closer detector placement within the microscope and more efficient light collection from highly scattering cells and tissues [19]. In traditional one-photon excitation microscopy (i.e., confocal microscopy), a confocal pinhole is used to spatially filter all emission that does not originate in the focal plane [19, 20]. In multiphoton microscopy, excitation occurs only in a very restricted spatial focus. Therefore, fluorescent light originates only from a specific focal plane, making the confocal pinhole unnecessary. Because the confocal pinhole aperture is no longer needed, non-descanned detectors can be used. These detectors are placed close to the sample, and the light does not have to pass through all the elements in a traditional microscopy system, which eliminates the risk of out-of-plane photodamage. One of the major challenges with intensity-based measurements of NAD(P)H and FAD is that they have a quantum yield that is an order of magnitude lower than other commonly used fluorophores such as fluorescein (~ 0.9) [19, 24, 25]. The measurement of the relatively weak fluorescence of NAD(P)H and FAD can be challenging in highly perfused tissues since hemodynamic changes can affect intensity-based measurements such as the optical redox ratio due to hemoglobin absorption [19, 25].

Fluorescence lifetime microscopy (FLIM) can overcome some of these challenges because it can extract metabolic information using one excitation wavelength, is independent of intensity, and is sensitive to the molecular environment [19, 26]. FLIM is typically used to determine the binding fraction of fluorophores based on their lifetimes in unbound and protein-bound states [19, 27]. NAD(P)H FLIM measurements can be an alternative to the optical redox ratio. Spatially and temporally resolved measurements of NAD(P)H are typically performed using time-correlated single-photon counting (TCSPC). This technique creates a histogram of lifetimes by measuring the time between a laser pulse and the detection of a single emitted photon. To analyze this type of data, biexponential models are commonly fitted to the histogram at each pixel to obtain the decay curve (Eq. 1)

1 $$I(t)={I}_0\left({A}_{1}e\left({}^{-t}\!\left/ \!{}_{{\tau}_1}\right.\right)+{A}_{2}e\left({}^{-t}\!\left/ \!{}_{{\tau}_2}\right.\right)\right.,$$

where *I*_0_ is the initial fluorescence intensity, *τ*_1_ and *τ*_2_ are the short and long lifetime components, respectively, and *A*_1_ and *A*_2_ are their respective relative contributions to the total fluorescence [19, 28]. The ratio of *A*_1_ / *A*_2_ is frequently used as a summary statistic to describe the lifetime decay of NADH [19, 28]. Overall, the use of the optical redox ratio and FLIM can be useful methods to assess metabolic and functional cellular changes across a broad spectrum of biomedical applications, including cancer development [19].

Current studies have been able to demonstrate in-depth metabolic characterization of macrophage populations in the two traditional subsets of macrophages (M1 and M2) after cytokine stimulation using optical advances such as multiphoton microscopy and fluorescence lifetime imaging (FLIM). The detection and quantification of the metabolic coenzymes NAD(P)H and FAD can provide an estimate of metabolic activity since metabolic potential coincides with the ratio of reduced and oxidized metabolic substrates. This enables an improved understanding of the heterogeneity of dynamic changes in redox states within cells, providing insight into macrophage metabolic reprogramming previously unachieved through other methods. However, the detection of NAD(P)H and FAD can be affected by factors other than metabolic changes (i.e., microenvironmental pH). Therefore, there is a need to validate optical measures of metabolism through autofluorescence imaging. In this study, we seek to validate optical measures of metabolism (i.e., optical redox ratio, mean NADH lifetime, and A1/A2 ratio) by comparing optical results to conventional methods of evaluating cellular metabolism, such as extracellular flux assays and detection of metabolic intermediates such as succinate and lactate.

## Materials/methods

### Macrophage culture

**Fig. 2:**
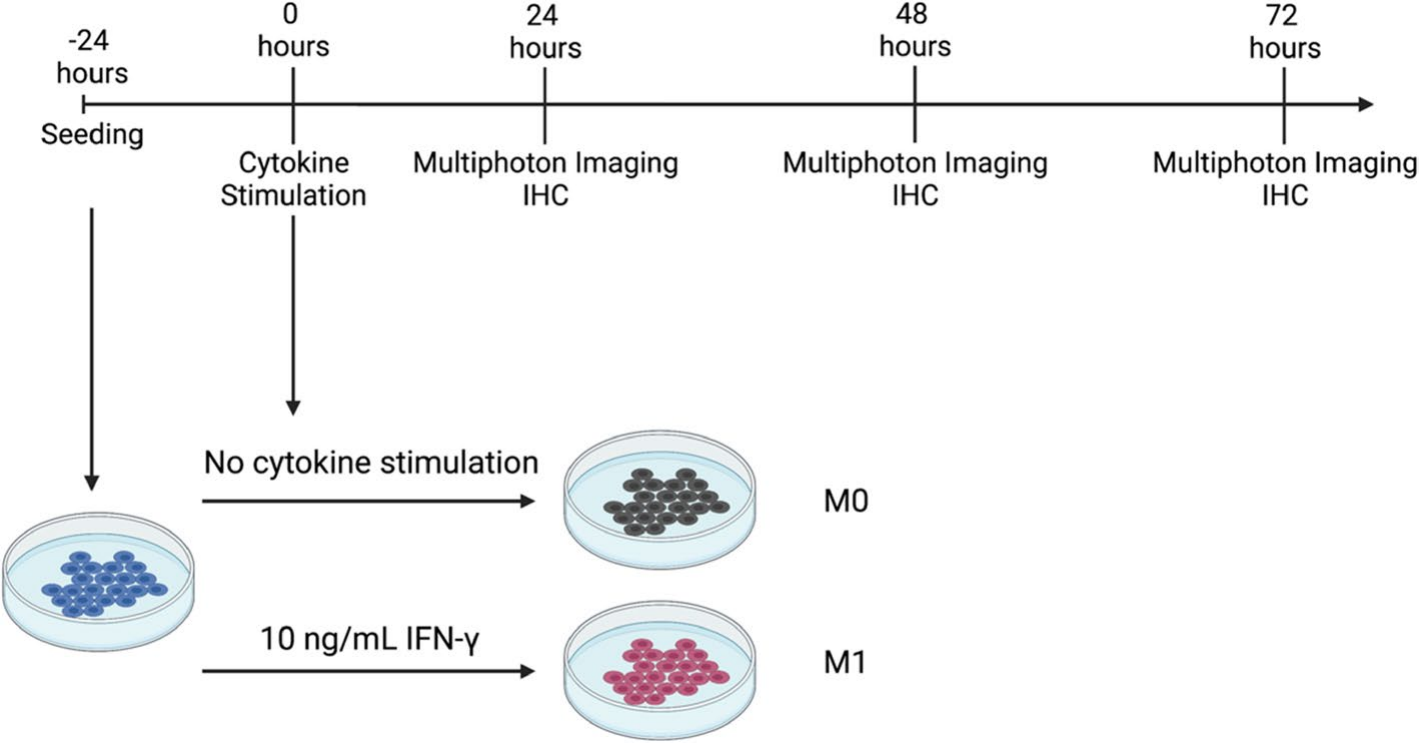
Schematic of RAW 264.7 macrophage differentiation

Murine RAW 264.7 (ATCC©, TIB-71™) macrophages were maintained in Roswell Park Memorial Institute (RPMI)-1640 medium (Corning™, 10104-CV) with 10% fetal bovine serum (FBS) (ATCC©, 30–2020™) and 1% gentamicin (Gibco™, 15710064). RAW 264.7 macrophages were seeded at a density of 1 × 10^6^ cells/mL in a 35 mm MatTek© culture dish at 37 °C and 5% CO_2_ 24 hours prior to cytokine stimulation to allow for cell adhesion to the culture dish. After the initial 24-hour (0-hour) period, macrophages were polarized towards the M0 or M1 phenotypes with the following: M0 (RPMI-1640 medium only) and M1 (RPMI-1640 medium supplemented with 10 ng/mL IFN-γ (R&D Systems®, 485-MI-100)) and allowed to incubate for an additional 24–72 hours (Fig. 2).

### Confirmation of macrophage differentiation

To confirm the successful differentiation of macrophages to their respective M0 and M1 populations, immunofluorescence staining was performed. A Brilliant Violet 421™ CD80 primary antibody was used to detect the surface expression of CD80, a common M1 marker, while an AlexaFluor 594 anti-mouse CD206 primary antibody was used to detect the surface expression of CD206, a common M2 marker. Images were acquired using an inverted laser scanning confocal microscope (Olympus Fluoview FV10i-LiV) with a 60X (1.2 N.A., water immersion) objective. Images were acquired 24 hours after initial cell seeding (0 hours) and at 24-hour intervals post-cytokine stimulation (24 hours, 48 hours, and 72 hours) for each culture condition.

### Live cell metabolic imaging

**Fig. 3:**
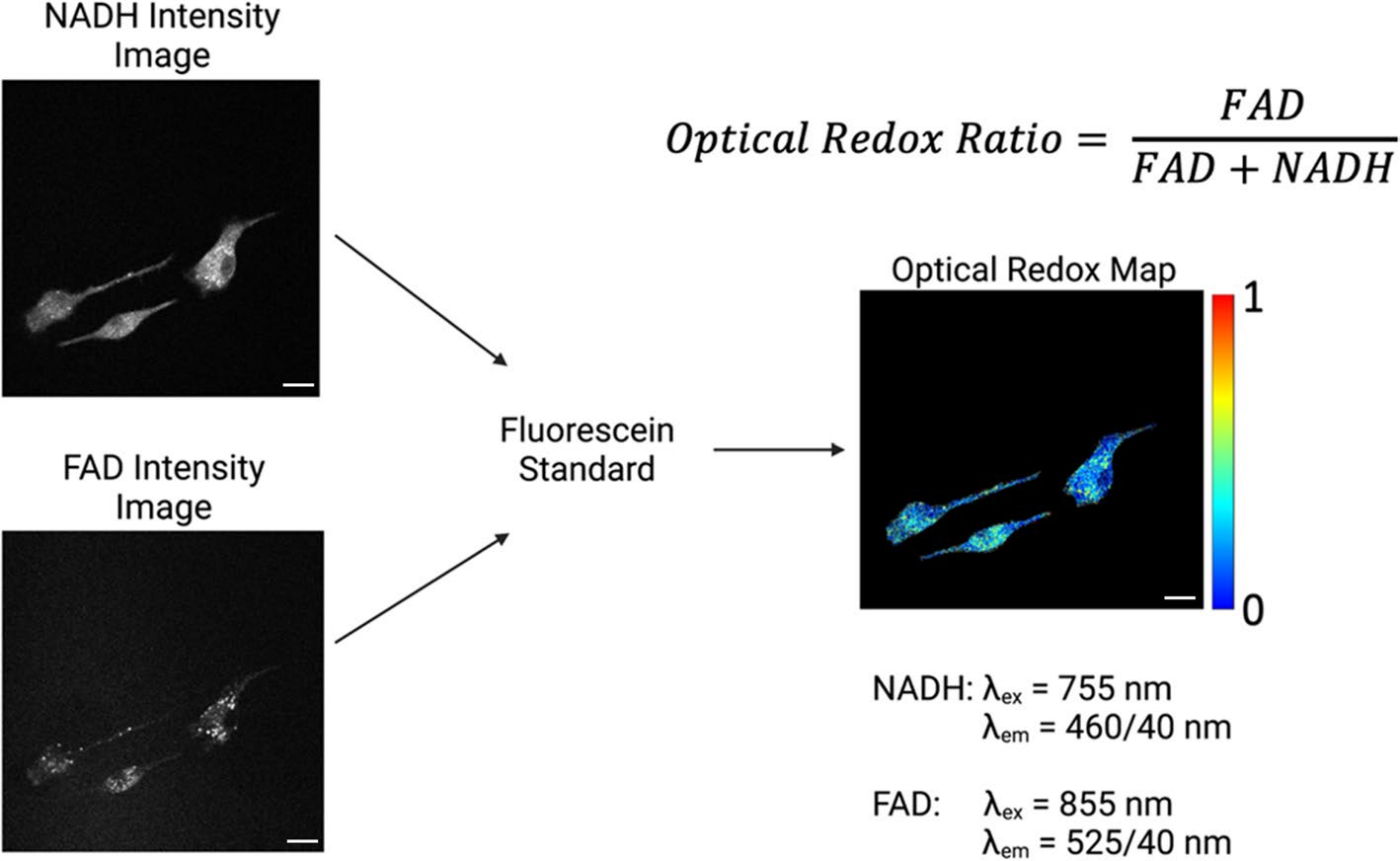
Flow diagram demonstrating the normalization of NADH and FAD intensity images using fluorescein and creating an optical redox map to calculate the optical redox ratio of individual cells. Scale bars are 20 μm. Figure was created with BioRender

Prior to imaging, cells were moved to a microincubator with a controllable temperature and humidified gas delivery (5% CO2). A custom inverted multiphoton imaging system (Bruker custom system) equipped with an Ultrafast Ti:Sapphire (Mai Tai HP, Spectra Physics, Inc.) via a (60x/1.2NA) water immersion objective (Olympus) and four close-proximity, high-efficiency GaAsP detectors. NADH fluorescence was captured with a 460 (± 20) nm bandpass filter at 755 nm excitation and FAD fluorescence with a 525 (± 25) nm bandpass filter at 855 nm excitation. NADH and FAD fluorescence were normalized by PMT gain and laser power, with PMT gain normalized to fluorescein concentrations (0.1 μM to 20 μM in Tris Buffer of pH 8) as in previous studies [29, 30]. PMT gain and laser power were kept constant, and laser power was read after each imaging session (Fig. 3). An integrated fluorescence lifetime imaging microscopy (FLIM) module was used to measure mitochondrial function regarding the different components contributing to NADH autofluorescence. Images were acquired 24 hours after initial cell seeding (0 hours) and at 24-hour intervals post-cytokine stimulation (24 hours, 48 hours, and 72 hours) for each culture condition.

### Metabolic intermediate detection

An important metabolic intermediate associated with glycolysis is the accumulation of succinate and increased lactate production. To quantitatively determine if there is a presence of succinate in the M0 and M1 macrophages, a colorimetric succinate assay was used. A standard curve was prepared using a 1 mM succinate standard. Differentiated macrophages (M0 or M1) were harvested at a density of 1 × 10^6^ cells. Cells were resuspended in 100 μL of ice-cold Succinate Assay Buffer. The supernatant was collected, transferred to a clean microcentrifuge tube, and kept on ice. After the standard and sample wells has been prepped using a 96-well plate, the well plate was transferred to a microplate reader to read the output (OD = 450 nm). The concentration of succinate in the samples was calculated using Eq. 2:

2 $$Succinate\ (mM)=\left(\frac{A}{B}\right)* D,$$

where A is the amount of succinate from the standard curve, B is the sample volume added into the reaction well, and D is the sample dilution factor.

To validate the presence of lactate production, a lactate assay kit was used to quantify where lactate is oxidized by lactate dehydrogenase to generate a product that interacts with a probe to produce a color. Briefly, differentiated macrophages (M0 or M1) were harvested at a density of 2 × 10^6^ cells and resuspended in lactate assay buffer. The supernatant was collected, transferred to a clean microcentrifuge tube and kept on ice prior to the start of the assay. The output was measured using a microplate reader at OD 450 nm. The lactate concentration was calculated using Eq. 3:

3 $$Lactate\ concentration=\left(\frac{La}{Sv}\right)\ast D,$$

where La is the amount of lactic acid in the sample well calculated from the standard curve, Sv is the volume of sample added to the well (μL) and D is the sample dilution factor.

### Oxygen consumption rate

A Seahorse XFp extracellular flux analyzer was used to establish the cellular measures of mitochondrial respiration using the oxygen consumption rate (an indicator of oxidative phosphorylation) of macrophages as they undergo activation [31]. Cell densities were optimized to ensure that cells were not over-confluent after cytokine stimulation. After cytokine stimulation, the cell media was replaced with Seahorse assay media and placed in a non-CO2 incubator at 37 °C for 60 minutes prior to the start of the assay. To calculate the oxygen consumption rate (OCR) and proton production rate (PPR), three compounds were added sequentially to perturb mitochondrial respiration – oligomycin (1.5 μM), carbonylcyanide p-triflouromethoxyphenylhydrazone (FCCP; 1.0 μM) and rotenone/antimycin A mixture (RAAM; 1.0 μM). Sequential injections of the drugs provided a list of other readouts (maximal respiration, ATP production, and spare respiratory capacity).

### Data analysis and statistics

Pixel-wise calculations of the optical redox ratio were computed in a custom MATLAB program after normalizing fluorescent intensities based on the day-to-day variability of the lasers [30]. A bi-exponential fit of the fluorescence lifetime decay using SPCImage provided the relative contribution of free (A1) and protein-bound (A2) NADH at each pixel. Pixel-wise mean fluorescence lifetime values, optical redox ratios, and the ratio of A1/A2 were calculated using a custom MATLAB program [30]. A mixed effects, one-way analysis of variance (ANOVA), and a Tukey’s honestly significant difference (HSD) test were used to evaluate statistical significance between specific experimental groups [30]. Macrophage cell lines and cytokine stimulation are considered fixed effects, and the fields of view within each group were considered random effects. A *p*-value of < 0.05 is considered statistically significant.

## Results

### Immunohistochemistry demonstrates plasticity in M1/M2 macrophage surface markers after cytokine stimulation of murine macrophages

**Fig. 4:**
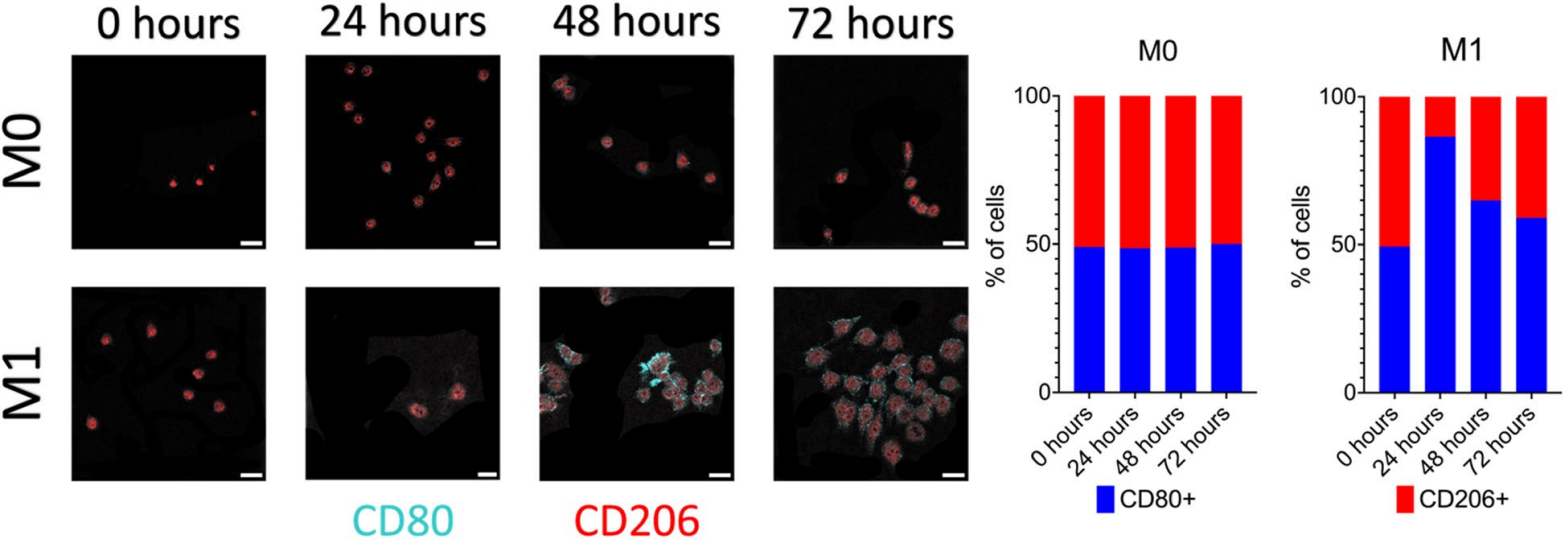
Macrophage phenotypic plasticity is demonstrated in immunohistochemistry of cytokine-stimulated murine macrophages. Representative immunofluorescence images show surface expression of common M1-like (CD80) and M2-like (CD206). Scale bar is 10 μm

The immunofluorescence of a common M1 (CD80) and CD206 (M2) surface marker was used to observe phenotypic changes as a macrophage underwent differentiation. As shown in Fig. 4, M0 macrophages show a rounded morphology during the 0- and 24-hour time periods, with the incorporation of elongated morphologies starting at 48 hours post-cytokine stimulation. Over time, there were no significant changes in CD80 and CD206 expression (~ 50% CD80 and ~ 50% CD206 expression). M1 macrophages show a shift from a rounded morphology to a more flattened, cuboidal structure during differentiation. As expected, M1 macrophages show a spike in the number of cells expressing CD80 after 24 hours (~ 85% CD80 and ~ 15% CD206). Interestingly, CD80 surface expression in M1 macrophages decreases over time and is approaching similar levels to those of pre-differentiation (0 hours). Overall, immunohistochemistry of common M1 and M2 surface markers reveals phenotypic plasticity in cytokine-stimulated macrophages.

### Measures of mitochondrial respiration show functional changes in macrophage metabolism during activation

**Fig. 5:**
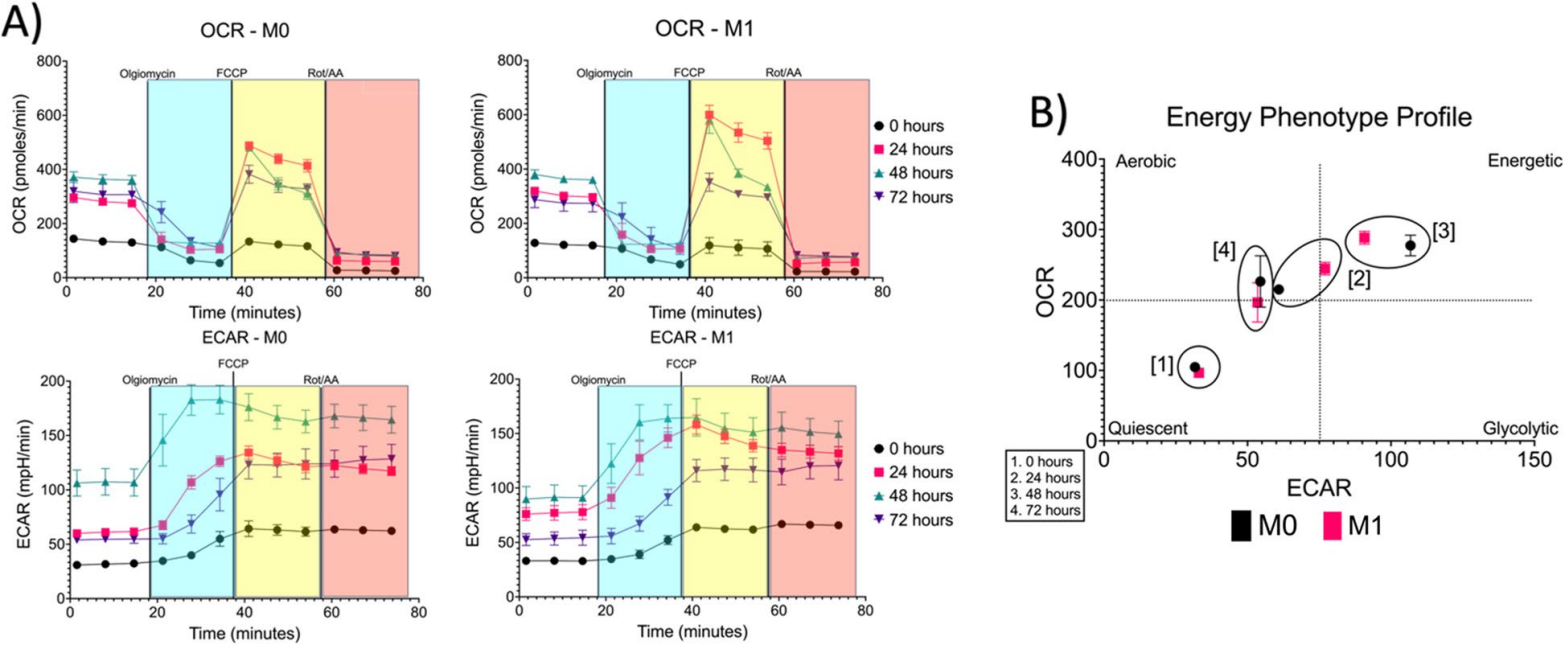
Data summary of Seahorse XFp Cell Mito Stress Test. **A** Profiles of Mito Stress Test data for OCR and ECAR (*n* = 3, M0; *n* = 3, M1). Shaded regions indicate which complexes of the electron transport chain are affected due to modulators: Blue (Complex IV), Yellow (Proton Gradient), and Red (Rotenone: Complex 1 and Antimycin A: Complex III). **B** Energy phenotype profiles generated for M0 and M1 macrophages based on basal OCR and ECAR levels for all timepoints (1: 0 hours, 2: 24 hours, 3: 48 hours, and 4: 72 hours). * *p* ≤ 0.05; ** *p* ≤ 0.01; *** *p* ≤ 0.001; **** *p* ≤ 0.0001. Graphs were made in GraphPad Prism®

Functional mitochondrial measurements are essential in understanding changes in cellular metabolism during activation, proliferation, differentiation, and dysfunction. Using the Seahorse XF Cell Mito Stress Test, we can obtain direct measurements of OCR while assessing key parameters of mitochondrial function. As shown in Fig. 5A, metabolic profiles of the OCR and ECAR were obtained. Over time, M0 macrophages show an increase in OCR and ECAR compared to the 0-hour time point. Similar trends can be observed in M1 macrophages, which have a higher OCR value at 24 hours compared to M0 macrophages. M1 macrophages also showed an increase in ECAR over time, with the 24- and 48-hour time points showing similar values. An energy phenotype profile for M0 and M1 macrophages was created by plotting basal ECAR and OCR levels to provide a snapshot of metabolic profiles during activation (Fig. 5B). Briefly, there are four relative bioenergetic phenotypes that a cell can display: quiescent (not energetic through glycolysis or mitochondrial respiration), energetic (utilizes glycolysis and mitochondrial respiration), aerobic (utilizes mitochondrial respiration), or glycolytic (utilizes glycolysis). Over time, M0 macrophages transitioned from a quiescent state (0 hours) to an aerobic state (24 hours), to an energetic state (48 hours), and back to an aerobic state (72 hours). Interestingly, M1 macrophages displayed a change from a quiescent state (0 hours) to an energetic state (24–48 hours) to an aerobic/quiescent state (72 hours). In Supplemental Fig. 1, key metabolic parameters were obtained from the OCR profile. For all parameters, M0 and M1 macrophages showed similar trends over time, apart from a small increase in H+ proton leak for M0 macrophages between the 24- and 48-hour timepoints and a decrease in H+ proton leak between the 24- and 48-hour timepoints. Since all parameters were significantly different from the 0-hour timepoint (Non-Mitochondrial Respiration, Maximal Respiration, Basal Respiration, ATP Production: *p* < 0.0001; H+ proton leak: M0: *p* = 0.0149, *p* = 0.085, M1: *p* < 0.0001, *p* = 0.0002; Coupling Efficiency: M0: *p* = 0.0484, *p* = 0.0018, *p* = 0.0003, M1: *p* = 0.0053, *p* = 0.0006; Spare Respiratory Capacity: M0: *p* < 0.0001, *p* = 0.0025, M1: *p* < 0.0001), it can be inferred that metabolism is actively occurring within the macrophages during activation.

**Fig. 6:**
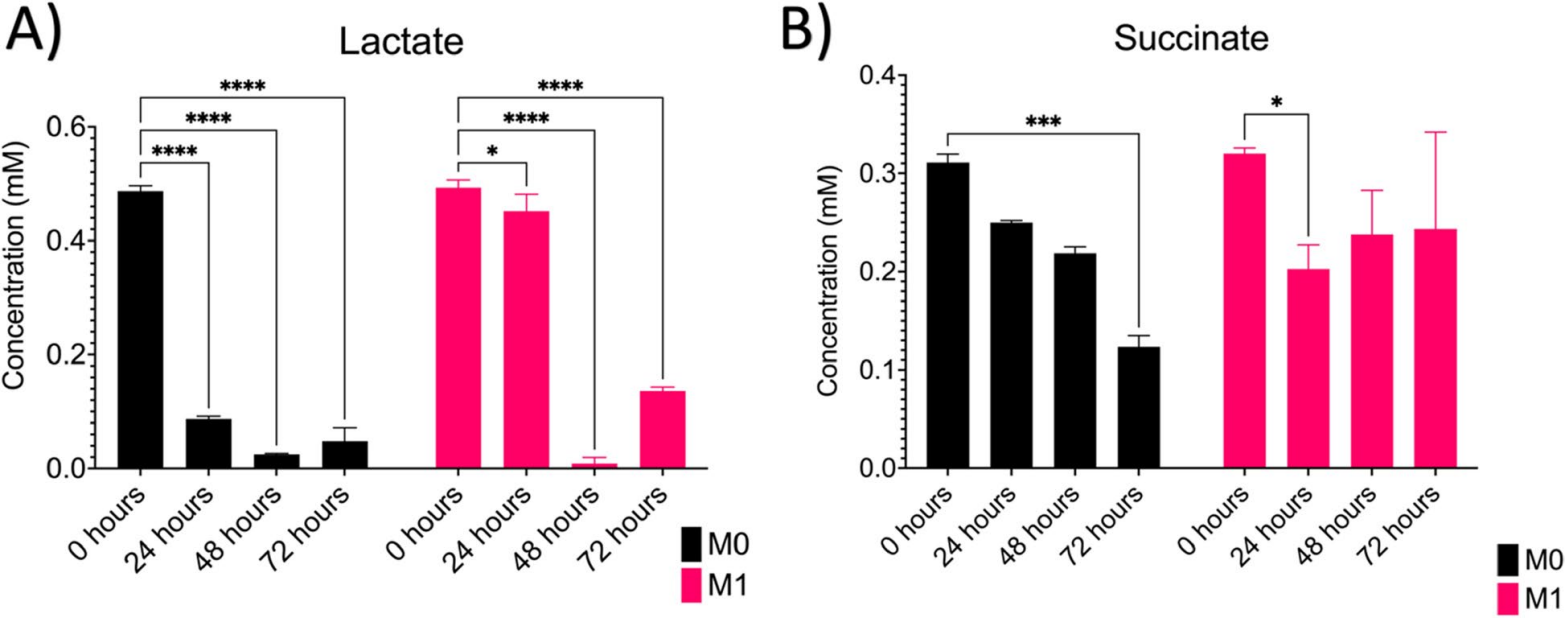
Data summary of colorimetric lactate and succinate assays. **A** Lactate and **B** Succinate concentration of macrophages over time (*n* = 3, M0; *n* = 3, M1). * *p* ≤ 0.05; ** *p* ≤ 0.01; *** *p* ≤ 0.001; **** *p* ≤ 0.0001. Graphs were made in GraphPad Prism®

In addition to investigating the metabolic profiles of cytokine-stimulated macrophages, we investigated the production of two characteristic metabolic intermediates used in inflammatory macrophage metabolism: lactate and succinate. As shown in Fig. 6A, M0 macrophages show a significant decrease in lactate production (~ 0.4 mM) across all time points (*p* < 0.0001). M1 macrophages showed a small but significant decrease in lactate production between 0 and 24 hours (~ 0.04 μM, *p* = 0.023) before a significant decrease in lactate production (~ 0.49 mM) after 48 hours (*p* < 0.0001). In Fig. 6B, M0 macrophages show a decrease in succinate production for the first 48 hours before decreasing significantly (~ 0.19 mM) after 72 hours (*p* = 0.0002). M1 macrophages show a significant decrease in succinate production (~ 0.12 mM) after 24 hours (*p* = 0.0114) before increasing (~ 0.08 mM) after 48 and 72 hours. Overall, our results indicate that M0 macrophages begin to utilize aerobic metabolism over time, while M1 macrophages show an indication of an increase in aerobic metabolism during the first 24 hours with a metabolic switch towards a quiescent/glycolytic state after 48 hours.

### Live cell metabolic imaging shows acute changes in endogenous NADH and FAD as they undergo activation

**Fig. 7:**
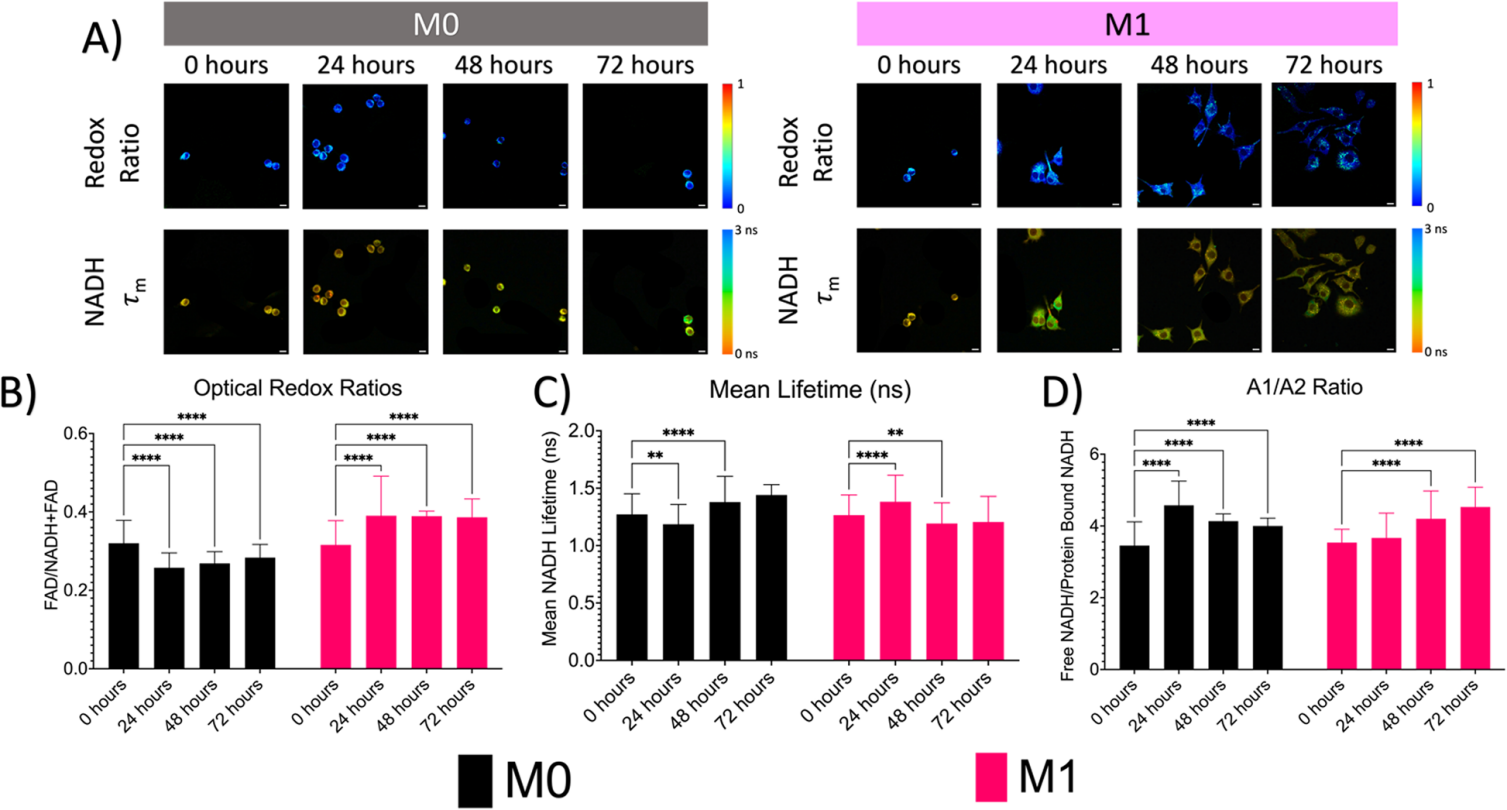
Autofluorescence imaging shows significant changes in cellular metabolism during macrophage activation after cytokine-stimulation. **A**) Representative optical redox (FAD/FAD+NADH) and mean NADH lifetime maps of M0 and M1 macrophages. Scale bars are 10 μm. **B**) Changes in optical redox ratios of M0 and M1 macrophages before and after cytokine stimulation. Changes in **C**) mean NADH lifetime (ns) and **D**) A1/A2 ratio over time. ** *p* ≤ 0.01; **** *p* ≤ 0.0001; Graphs were made in GraphPad Prism®

To establish the sensitivity of metabolic autofluorescence imaging to longitudinally track macrophage differentiation, metabolic autofluorescence measurements were acquired in monolayer cultures of cytokine-stimulated RAW 264.7 macrophages. Macrophages were stimulated with IFN-𝛾 (M1) or without cytokine stimulation (M0) and maintained and imaged over 24–72 hours. As shown in Fig. 7A, autofluorescence imaging showed similar changes in morphology that were observed in autofluorescence imaging. Autofluorescence images of NADH and FAD show acute, time-dependent changes between the M0 and M1 phenotypes. In Fig. 7B, M0 macrophages show a significant decrease (0.320 to 0.258) in the optical redox ratio after 24 hours (*p* < 0.0001), with a slow increase at the 48-hour (*p* < 0.0001) and 72-hour (*p* < 0.0001) time periods. M1 macrophages show a significant increase in optical redox ratio (0.316 to 0.390) after 24 hours (*p* < 0.0001) before maintaining a consistent redox ratio (~ 0.386) after 48 hours (*p* < 0.0001).

Autofluorescence intensity images can provide information on the spatial distribution of endogenous fluorophores and can discriminate between those fluorophores with distinct spectral properties. However, intensity images alone cannot distinguish fluorophores with similar spectra or the microenvironment around those fluorophores [32]. FLIM offers this advantage over intensity-based autofluorescence imaging. Overall, small changes in mean NADH lifetime were observed in both M0 and M1 phenotypes (Fig. 7C**)**. More specifically, a small but significant decrease (1.272 ns to 1.186 ns) was observed in M0 macrophages after 24 hours (*p* = 0.0012) before increasing (1.379 ns) after 48 hours (*p* < 0.0001). M1 macrophages show a significant increase (1.265 ns to 1.382 ns) after 24 hours (*p* < 0.0001) before approaching baseline levels after 72 hours (1.206 ns). Additional analysis of NADH lifetime data through a bi-exponential decay curve can provide the short (free NADH, A1) and long (protein-bound NADH, A2) components as their respective contributions to the total fluorescence [19]. The ratio of A1/A2 is a common summary statistic to describe the lifetime decay of NADH. As shown in Fig. 5D, M0 macrophages display a significant increase (3.452 to 4.576) in the A1/A2 ratio after 24 hours, (*p* < 0.0001) with a slow decline over time (*p* < 0.0001). M1 macrophages show a slight increase (3.537 to 3.66) in the A1/A2 ratio after 24 hours with a significantly steady (4.202 and 4.529, respectively) increase over time (*p* < 0.0001). Based on these trends, M0 macrophages are favoring an aerobic metabolism over time, while M1 macrophages show a trend towards mitochondrial respiration within the first 24 hours before becoming more glycolytic over time. Overall, our results also indicate that optical redox mapping and FLIM can show discrete changes in NADH within the microenvironment and the macrophage cytoplasm as they undergo activation post-cytokine stimulation.

### Frequency distributions show macrophage heterogeneity within M0 and M1 macrophage populations

**Fig. 8:**
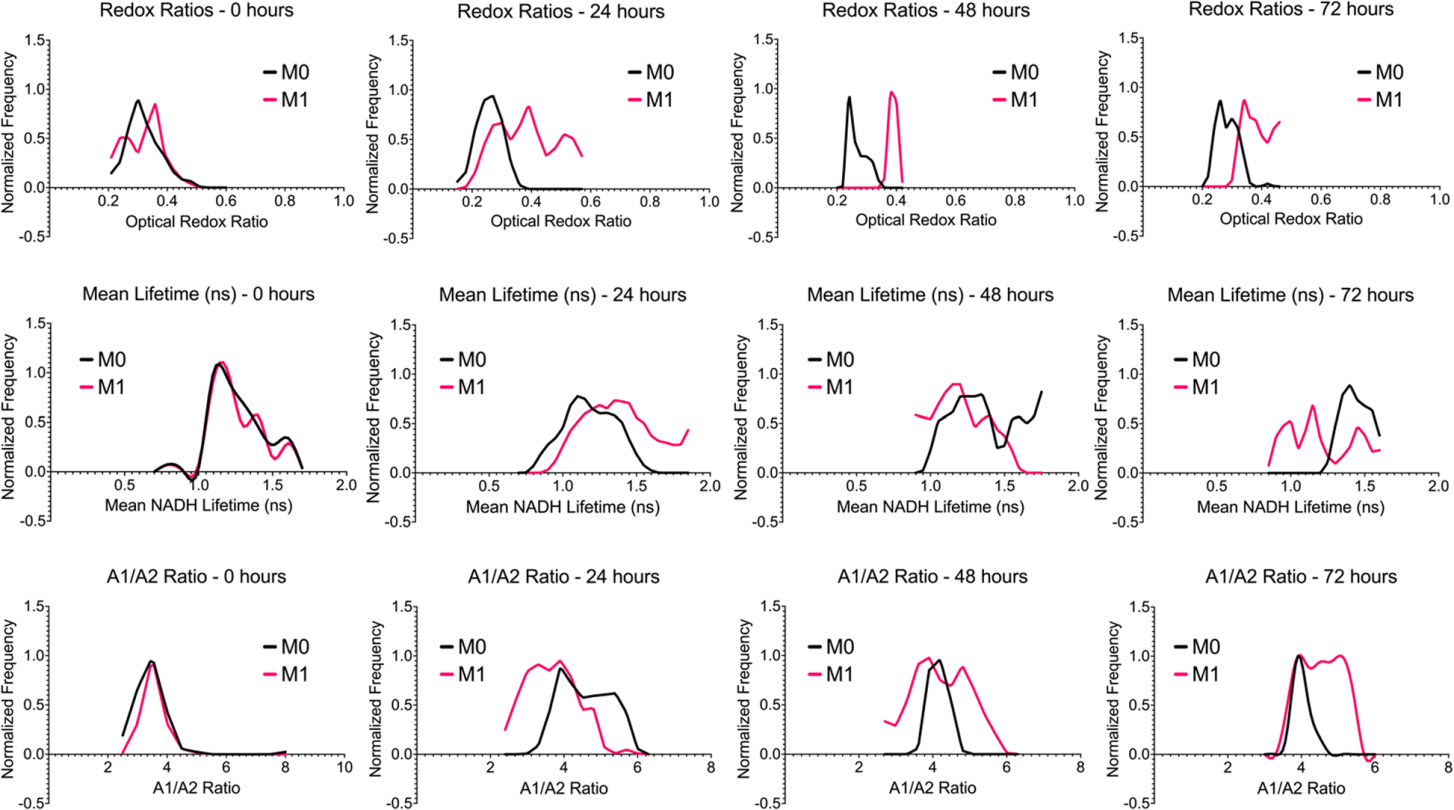
Normalized Frequency distribution curves of show subpopulations of macrophages within M0 and M1 phenotypes. Top: Optical redox ratio. Middle: Mean NADH Lifetime (ns). Bottom: A1/A2 Ratio. Frequency distribution curves were created in GraphPad Prism®

Macrophages have been traditionally classified into the M1 and M2 phenotypes. However, recent studies are now showing that macrophages rarely display one distinct phenotype in tissue, which makes them highly plastic in nature. This plasticity allows them to change their physiology in response to various environmental stimuli, allowing them to give rise to various populations with separate functions [33]. Based on this, further analysis through normalized frequency distribution curves of our optical data was performed to investigate heterogeneity within aggregated cell data. Frequency distribution curves revealed heterogeneous metabolic subpopulations (*n* = 100) across all conditions and timepoints (Fig. 8). For the optical redox ratio, the majority of the M0 macrophage population lies between 0.2 and 0.4, with a single peak around 0.25 across all time points. M1 macrophages, on the other hand, showed broader ranges of redox ratio values for the 0-hour (0.2 to 0.5), 24-hour (0.2 to 0.6), and 72-hour (0.2 to 0.5) timepoints. These timepoints also showed more distinct peaks than the M0 macrophages. The 0-hour timepoint showed two peaks at 0.27 and 0.36, while the 24-hour timepoint showed three peaks at 0.30, 0.39, and 0.51. The 48-hour timepoint showed one singular peak at 0.38, while the 72-hour timepoint showed a peak at 0.34. When comparing trends over time, a shift in the optical redox ratio for M1 macrophages can be seen, whereas the optical redox ratio for M0 macrophages remains stable.

Moving to mean NADH lifetime, M0 and M1 macrophages show a broader range of values across all timepoints (0.7 to 1.7 ns, 0.7 to 1.85 ns, 0.9 to 1.75 ns, and 0.85 to 1.6 ns). At the 0-hour timepoint, one distinct peak was observed at 1.10 ns, with a smaller peak around 1.6 ns. For the 24-hour timepoint, only one peak was observed at 1.1 ns, while the 48-hour timepoint showed three peaks at 1.2 ns, 1.55 ns, and 1.75 ns. The 72-hour timepoint showed one peak at 1.4 ns. For M1 macrophages, the 0-hour timepoint showed three peaks at 1.2 ns, 1.4 ns, and 1.6 ns. At 24 hours, no distinct peaks were observed, but in the 1.1 to 1.6 ns range, the frequency ranges between 0.45 and 1.0. At 48 hours, two peaks were observed at 1.2 ns and 1.4 ns. Lastly, for the 72-hour timepoint, three peaks were observed at 1.0 ns, 1.15 ns, and 1.45 ns.

Lastly, we investigated the frequency distributions in the A1/A2 ratio. At 0 hours, M0 macrophages showed a range of values between 2.5 and 8.0, while M1 macrophages showed a range between 2.5 and 5. For both phenotypes, one peak was observed at a value of 3.5. After 24 hours, M0 macrophages showed a range between 3.3 and 5.7, while M1 macrophages showed a range of values between 2.4 and 4.8. M0 macrophages showed one distinct peak at 3.9. However, in the range of values from 4.2 to 5.7, the frequency ranges between 0.45 and 0.70. M1 macrophages did not show a distinct peak, but in the range of values from 3.0 to 4.2, the frequency ranges between 0.81 and 1.00. At 48 hours, M0 macrophages show a range of values between 3.9 and 4.5 with one distinct peak at 4.2, while M1 macrophages show a range of values between 2.7 and 5.7 with two distinct peaks at 3.9 and 4.8. After 72 hours, M0 macrophages show a range of values between 3.6 and 4.5 with one peak at 3.9, while M1 macrophages show a range of values between 3.6 and 5.4 with no distinct peaks but a frequency that ranges between 0.882 and 1.00 between the values of 3.9 and 5.1. Results could indicate that optical imaging through autofluorescence of endogenous metabolites can show how cellular subpopulations can change over time based on cytoplasmic and environmental measurements of metabolism.

### Correlation between autofluorescence imaging and metabolism assays

To investigate whether there are correlations between autofluorescence imaging and traditional metabolic assays, correlation plots were created. First, we compared lactate concentration to key OCR parameters (Supplemental Fig. 2). Four significant correlations were observed: lactate concentration vs. non-mitochondrial respiration (*p* = 0.002, Pearson’s *r* = − 0.9047), lactate concentration vs. basal respiration (*p* = 0.0307, Pearson’s *r* = − 0.7539), lactate concentration vs. ATP production (*p* = 0.0163, Pearson’s *r* = − 0.8033), and lactate concentration vs. coupling efficiency (*p* = 0.0039, Pearson’s *r* = − 0.8805). For all other OCR parameters, wide 95% confidence intervals were observed.

Next, we compared succinate concentration to key OCR parameters (Supplemental Fig. 3). Here, we see three significant correlations: succinate concentration vs. non-mitochondrial respiration (*p* = 0.0273, Pearson’s *r* = − 0.7641), succinate concentration vs. ATP production (*p* = 0.0434, Pearson’s *r* = − 0.7213), and succinate concentration vs. coupling efficiency (*p* = 0.0183, Pearson’s *r* = − 0.7952). There were two other correlations that were close to being significant: succinate concentration vs. basal respiration (*p* = 0.0609, Pearson’s *r* = − 0.6848) and succinate concentration vs. maximal respiration (*p* = 0.1616, Pearson’s *r* = − 0.5459).

Next, we compared optical imaging metrics (optical redox ratio, mean NADH lifetime, and A1/A2 ratio) with key OCR parameters (Supplemental Figs. 4–6). As shown in Supplemental Figs. 4 and 5, there were no statistically significant correlations between the optical redox ratio, mean NADH lifetime, and OCR parameters, and all parameters showed wide 95% confidence intervals with low Pearson’s r values. The correlations between A1/A2 ratio and OCR parameters did show two statistically significant correlations: A1/A2 ratio vs. non-mitochondrial respiration (*p* = 0.0354, Pearson’s *r* = 0.7410) and A1/A2 ratio vs. coupling efficiency (*p* = 0.0394, Pearson’s *r* = 0.7311). For all other OCR parameters, wide 95% confidence intervals were observed. However, two other correlations were almost statistically significant: the A1/A2 ratio vs. basal respiration (*p* = 0.1558, Pearson’s *r* = 0.5523) and the A1/A2 ratio vs. ATP production (*p* = 0.1265, Pearson’s *r* = 0.5864).

Lastly, we compared optical imaging metrics (optical redox ratio, mean NADH lifetime, and A1/A2 ratio) with lactate/succinate concentration (Supplemental Fig. 7). When comparing lactate concentration with optical imaging metrics, one statistically significant correlation was observed: A1/A2 ratio vs. lactate concentration (*p* = 0.0133, Pearson’s *r* = − 0.8171). Even though no significant correlations were observed when comparing succinate concentration with optical imaging metrics, one correlation was almost statistically significant: mean NADH lifetime vs. succinate concentration (*p* = 0.0865, Pearson’s *r* = − 0.6414). Overall, our results indicate that there are statistically significant correlations between autofluorescence imaging of cellular metabolism and traditional metabolic assays.

## Discussion

Macrophages are an abundant subset of immune cells that comprise the tumor microenvironment and can have a variety of functions depending on the cytokines released from surrounding cells and tissues [1]. Traditionally, macrophages are usually classified into two phenotypes: M1 (anti-tumor) and M2 (pro-tumor). However, recent studies have shown that macrophages rarely display one phenotype, making them highly plastic in nature based on their ability to change their physiology in response to environmental stimuli. Current studies have been able to study macrophage polarization through the measurement of cellular metabolism involved in metabolic reprogramming based on external stimuli. However, these studies only investigate metabolic readouts for bulk populations of cells within a sample, which limits metabolic information at the single-cell level [2, 4–7]. The use of optical imaging techniques such as multiphoton microscopy and fluorescence lifetime imaging are valuable tools that are emerging in the immunology field to provide spatial information on biological processes such as migration and activation using the metabolic co-factors NADH and FAD. The detection and quantification of these co-factors can give researchers a better understanding of the dynamic changes in the redox states within cells. However, the detection of NADH and FAD can be affected by microenvironmental factors (i.e., temperature, pH, and oxygen levels) as well as cytoplasmic metabolic changes. Therefore, there is a need to validate the detection of optical metabolic co-factors, NADH and FAD, in the cytoplasm. Here, we used autofluorescence imaging of endogenous metabolic co-factors, NADH and FAD, in conjunction with the assessment of oxygen consumption rate and metabolic intermediates to detect acute changes in cellular metabolism using optical redox states and the ratio of free to protein-bound NADH in quiescent and classically activated macrophage phenotypes, along with their intermediate states in response to targeted cytokine stimulation.

Macrophages are one of the most abundant immune cell populations within the tumor microenvironment and can express both anti-tumor and pro-tumor functions depending on the external stimuli which can affect their polarization towards an M1 (classical) or M2 (alternative) phenotype. To better understand the phenotypic changes of these macrophages after exposure to external stimuli, a direct immunocytochemistry protocol using two primary-conjugated macrophage antibodies, anti-CD80 (M1) and anti-CD206 (M2) was used. The use of immunofluorescence was used determine if 1) cytokine stimulation was successful in activating macrophages towards the correct phenotype and 2) detecting cellular heterogeneity within the cell population during activation. Based on the results, M0 macrophages transform from a rounded morphology to an elongated morphology with approximately 50/50 surface expression of common M1 (CD80) and M2 (CD206) markers. M1 macrophages showed a shift from a rounded morphology to a more cuboidal structure; however, over time, M1 macrophages showed a spike in CD80 surface expression during the first 24 hours before those levels decreased after 48 and 72 hours. However, these assays are limited to fixed cells, with the goal of observing structures within the cell and providing no spatial information on cellular function.

To better understand cellular physiology, an assessment of cellular energy metabolism is essential. Cells require energy in the form of ATP to support various functions such as proliferation, differentiation, etc. The study of cellular metabolism includes the biochemical pathways, intermediate metabolites, carbon sources, signaling networks, and regulatory mechanisms that control the intricate process of generating and consuming ATP [15, 16]. To gain a better understanding of how cellular metabolism changes in macrophages during activation, a Seahorse XFp extracellular flux analyzer was used to measure changes in OCR and ECAR. Our results show that although M0 and M1 macrophages show similar trends in their OCR and ECAR profiles, M0 macrophages show a transition from a quiescent energy state to a more aerobic state, which could indicate a shift towards OXPHOS. M1 macrophages displayed a transition to an energetic state during the first 48 hours, which indicates that they are using both glycolysis and oxidative phosphorylation to produce ATP. However, after 72 hours, M1 macrophages approach an aerobic/quiescent state, meaning that there is a reduction in OXPHOS.

Based on these trends, we further investigated whether metabolic intermediates (lactate and succinate) could influence the metabolic profiles in our macrophage model over time. Studies have shown that M1 macrophages are known to display a more glycolytic profile due to the increased production of lactate and accumulation of succinate due to a break in the TCA cycle [10]. Our results show that M0 macrophages show high levels of lactate production at 0 hours before a significant decrease in production after 24 hours. This could indicate that M0 macrophages are showing a shift away from glycolysis and towards oxidative phosphorylation due to the decrease in lactate production. Our results also show that M1 macrophages show a slight decrease in lactate production during the first 24 hours before significantly decreasing after 48 hours. This could indicate that M1 macrophages show an immediate shift towards glycolysis during the first 24 hours before shifting towards OXPHOS after 48 hours. Although these metabolic assays do provide information on changes in cellular metabolism, they only provide a bulk readout within a sample, which can obscure the effects of environmental variables on macrophages over time [34]. Therefore, understanding a cell’s metabolic state at the single-cell level can help researchers explain the molecular mechanisms underlying their phenotypic heterogeneity within a complex microenvironment.

Optical imaging of cellular autofluorescence is increasingly used as a tool to study cellular and tissue allowing for the longitudinal evaluation of biological processes [19, 21]. An advantage of optical imaging is the ability to resolve subcellular details using high-resolution microscopy techniques that can measure signals at specific wavelengths, which can provide specificity in exploring intrinsic fluorophores that can play a key role in cellular metabolism. Cellular redox networks that are responsible for controlling cellular metabolism can adapt to changes in the microenvironment to help maintain the single-cell and multicellular processes that keep the cell in homeostasis. Reliable endogenous indicators of cellular metabolism, such as pyridine nucleotides and flavoproteins, have been identified. More specifically, NAD can exist in a reduced (NADH), oxidized (NAD+), or phosphorylated form (NADPH). Similarly, flavin adenine dinucleotide (FAD) can also be in a reduced (FADH2) or oxidized (FAD) form. Since NADPH exhibits a higher fluorescence intensity than FAD within cells, the quantification of these autofluorescent signals can provide an estimate of metabolic activity due to the metabolic potential that coincides with the ratio of reduced and oxidized metabolic substrates [19, 23]. Therefore, the use of high-resolution microscopy can enable us to obtain insight into the heterogeneity of dynamic changes in cellular redox states. Here, we used two-photon microscopy and FLIM to investigate the optical redox states and the changes in free and protein-bound NADH in quiescent and classically activated macrophage phenotypes at the single-cell level and to determine if cellular heterogeneity within these subpopulations is present. First, we investigated changes in the optical redox ratio. We observed that M0 macrophages showed a significant decrease in optical redox ratio after 24 hours before slowly increasing after 48 hours. This could indicate a shift towards glycolysis (a decrease in the optical redox ratio) within the first 24 hours before a slow shift towards OXPHOS (an increase in the optical redox ratio). Further analysis using normalized frequency distributions showed that M0 macrophages maintained a consistent range of optical redox values (~ 0.2 to 0.4) with a single peak across all time points, indicating that M0 macrophages, based on optical redox ratios alone, show a more homogeneous population. M1 macrophages did show a significant increase in optical redox ratio during the first 24 hours before maintaining a consistent optical redox ratio after 48 hours. This could indicate a shift toward OXPHOS during the first 24 hours. Interestingly, M1 macrophages showed a broader range of optical redox ratios with an increase in peaks over time while showing a shift towards higher optical redox ratio values over time. Because these ratios are based on intensity-based measurements of NAD(P)H and FAD, their quantum yield is an order of magnitude lower than that of other common fluorophores, such as fluorescein. These relatively weak fluorescence measurements can be difficult due to hemodynamic changes within the tissue, which can affect intensity-based measurements [19, 24, 25].

To combat these challenges, FLIM can be used due to its ability to extract metabolic information using one excitation wavelength while being independent of intensity and being more sensitive to changes in the molecular environment, allowing it to be an alternative to the optical redox ratio [19, 26]. M0 macrophages showed a slight decrease in mean NADH lifetime after 24 hours before increasing over time, which could indicate a shift towards OXPHOS metabolism (an increase in mean NADH lifetime). Further analysis of normalized frequency distributions did show a broader range of mean NADH lifetime values, with the majority of cells falling within one distinct peak across all time points. M1 macrophages showed a spike in mean NADH lifetime before decreasing and approaching levels observed at the 0-hour timepoint. This could indicate an immediate shift in oxidative phosphorylation before shifting back to glycolysis (a decrease in mean NADH lifetime). Normalized frequency distributions show similar trends as M0 macrophages in broad ranges of mean NADH lifetime values, but with an increase in the number of peaks observed. This could indicate that M1 macrophages show more heterogeneity over time based on the mean NADH lifetime alone.

In addition to investigating the lifetime of NADH, additional analysis can be performed using a biexponential decay curve to provide the short (free NADH, A1) and long (protein-bound NADH, A2) components as their respective contributions to the total fluorescence, which can be converted into the A1/A2 ratio (a common summary statistic to describe the lifetime decay of NADH) [19, 28]. Our results show that M0 macrophages show a significant increase in the A1/A2 ratio after 24 hours before slowly declining over time, which could indicate a shift towards aerobic metabolism (a decrease in the A1/A2 ratio) after a spike in glycolysis (an increase in the A1/A2 ratio). The normalized frequency distributions of M0 macrophages showed a broad range of A1/A2 ratios over time, with distinct peaks being observed at the 0- and 48-hour timepoints. During the 24- and 72-hour time points, no distinct peaks were observed; however, located within a range of A1/A2 ratios, there were high ranges of frequency that varied from 0.45 to 1.00. This could indicate that cellular heterogeneity occurs during those time periods. M1 macrophages showed a steady increase in the A1/A2 ratio over time, indicating a shift toward glycolysis during activation. Like M0 macrophages, M1 macrophages showed a wide range of A1/A2 ratios over time, with distinct peaks being observed at the 0- and 48-hour time points. During the 24- and 72-hour time points, no distinct peaks were observed; however, located within a range of A1/A2 ratios, there were high ranges of frequency that varied from 0.81 to 1.00. Overall, our results show that optical redox mapping and changes in NADH within the macrophage cytoplasm can show acute changes in cellular metabolism and within the microenvironment during activation. In addition, our results also indicate that optical imaging can show how cellular heterogeneity can change over time based on microenvironmental measurements of metabolism.

Two-photon microscopy and FLIM are newly applied methods that are currently being used to capture the dynamic nature of immune cell behavior; however, the detection of metabolic co-factors can be affected by external microenvironmental factors (i.e., temperature, pH). Therefore, the validation of these optical measures is essential. Here, we examined whether correlation exists between gold standard metabolic assays (Seahorse), intracellular concentrations of metabolic intermediates (lactate and succinate), and autofluorescence imaging of metabolic cofactors. Our results show that as the lactate and succinate concentrations within a macrophage increase, the non-mitochondrial respiration and coupling efficiency of the macrophage decrease. During anaerobic glycolysis, glucose is converted to pyruvate and then to lactate, where there is a net production of two ATP molecules compared to the 36 ATP molecules that are generated from the TCA cycle [35]. Coupling efficiency is the fraction of mitochondrial respiration that is dedicated to ATP synthesis [35]. Due to the accumulation of lactate generated through anaerobic glycolysis, there is a decrease in the net ATP molecules that are produced compared to the basal respiration rate of the cell, which could affect the coupling efficiency [35]. Although not well defined, non-mitochondrial respiration is an oxygen-consuming process that originates from pro-inflammatory enzymes such as NADPH oxidases. NADPH oxidases are a family of enzymes that catalyze the transfer of electrons to oxygen by generating superoxide or ROS using NADPH as an electron donor [36]. Studies have previously shown that an increase in glycolysis shows an increase in the flux of glucose through the PPP, increasing the production of purines and pyrimidines, which can be used for biosynthesis within the cell. It can also provide NADPH for NADPH oxidase, leading to an increase in ROS. This indicates a metabolic shift in maturely activated immune cells [37]. An increase in glycolysis could be a mechanism to rapidly increase ATP production, even though it is inefficient compared to OXPHOS. Furthermore, in activated macrophages, mitochondrial ROS production is increased, serving as a mechanism for phagocytosis. ATP produced through glycolysis could compensate for the shift away from mitochondrial metabolism from ATP production to ROS production by the electron transport chain [37]. Therefore, based on these correlations, if a shift in lactate production increases, there could also be a shift towards ATP production in the cell rather than ROS production, which could also indicate whether a macrophage has become fully activated. It was also determined that as the A1/A2 ratio increased, the non-mitochondrial respiration and coupling efficiency of a macrophage increased. Lastly, although not statistically significant, results indicated that as succinate concentration increases, the mean NADH lifetime decreases. It is known that an accumulation of succinate within the cytoplasm is an indicator of glycolysis, as is a decrease in the mean NADH lifetime [19]. This could imply that a decrease in mean NADH lifetime could be an indicator of glycolytic metabolism. Overall, two-photon microscopy and FLIM could be a newly applied methodology that could be applied to the immunology field to help researchers investigate changes in immune cells during activation and possibly during important cellular functions such as phagocytosis.

## Conclusions

Overall, the results of this study indicate that autofluorescence imaging of endogenous metabolic cofactors can be a reliable method for monitoring the temporal and spatial dynamics of macrophage metabolism as they undergo activation after stimulation with cytokines. This imaging methodology also revealed metabolic heterogeneity within the M0 and M1 phenotypes based on metabolism that traditional bulk metabolic assays could not. This approach could be applied to future studies investigating metabolic heterogeneity in macrophages performing essential functions such as phagocytosis in traditional monolayer cultures and more complex 3D cultures. Ultimately, these optical tools could help immunologists improve their understanding of cellular and metabolic heterogeneity and how this could potentially affect disease progression and treatment response in diseases where macrophages are prevalent.

### Supplementary Information

**Additional file 1.**

## Abbreviations

2-NBDG: 2-deoxy-2-[(7-nitro-2,1,3-benzoxadiazol-4-yl) amino]-D-glucose
18F FDG: 2-[18F]-2-deoxy-D-glucose
𝛼KG: Alpha-ketoglutarate
ATP: Adenosine triphosphate
ECAR: Extracellular acidification rate
ETC: Electron transport chain
FAD: Flavin adenine dinucleotide
FLIM: Fluorescence lifetime imaging
G6P: Glucose 6-phosphate
GLUT: Glucose transporter
HIF-1𝛼: Hypoxia-inducible factor 1-alpha
IL: Interleukin
iNOS: Inducible nitric oxide synthase
MCT: Monocarboxylate transporters
NAD: Nicotinamide adenine dinucleotide
NADP: Nicotinamide adenine dinucleotide phosphate
NO: Nitric oxide
OCR: Oxygen consumption rate
OXPHOS: Oxidative phosphorylation
PET: Positron emission tomography
PHDs: Prolyl hydroxylases
PPP: Pentose phosphate pathway
ROS: Reactive oxygen species
SDH: Succinate dehydrogenase
TCA: Tricarboxylic acid
TCSPC: Time correlated single photon counting
UV: Ultraviolet
VEGF: Vascular endothelial growth factor
XF: Extracellular flux
