# Supplementary material for: Autofluorescence imaging of endogenous metabolic cofactors in response to cytokine stimulation of classically activated macrophages
Source: Cancer Metab. 2023 Nov 13;11:22. doi: 10.1186/s40170-023-00325-z (PMC10644562; doi:10.1186/s40170-023-00325-z)
Supplement: Supplementary file 1 — Additional file 1. [file 40170_2023_325_MOESM1_ESM.docx]

**Autofluorescence Imaging of Endogenous Metabolic Cofactors in Response to Cytokine Stimulation of Classically Activated Macrophages – Supplemental Information**

Shelby N. Bess^1^, Matthew J. Igoe^1^, Abby C. Denison^1^, Timothy J. Muldoon^1*^

^1^Department of Biomedical Engineering, University of Arkansas, Fayetteville, AR, USA

* Contact information: tmuldoon@uark.edu


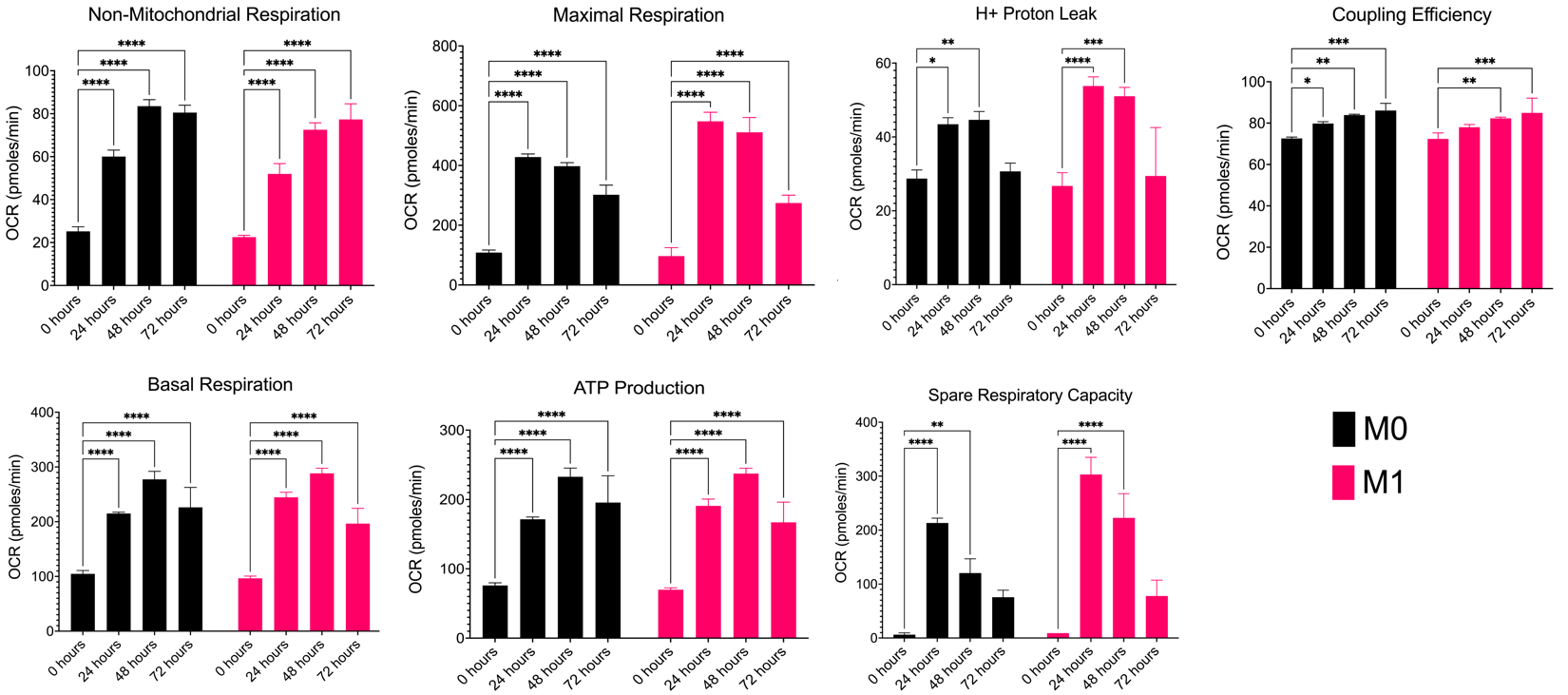


**Supplemental Figure 1.  Absolute OCR values calculated from Seahorse XFp Cell Mito Stress.** Absolute values for key OCR parameters over time (n = 3, M0; n = 3, M1). * p ≤ 0.05; ** p ≤ 0.01; *** p ≤ 0.001; **** p ≤ 0.0001. Graphs were made in GraphPad Prism®


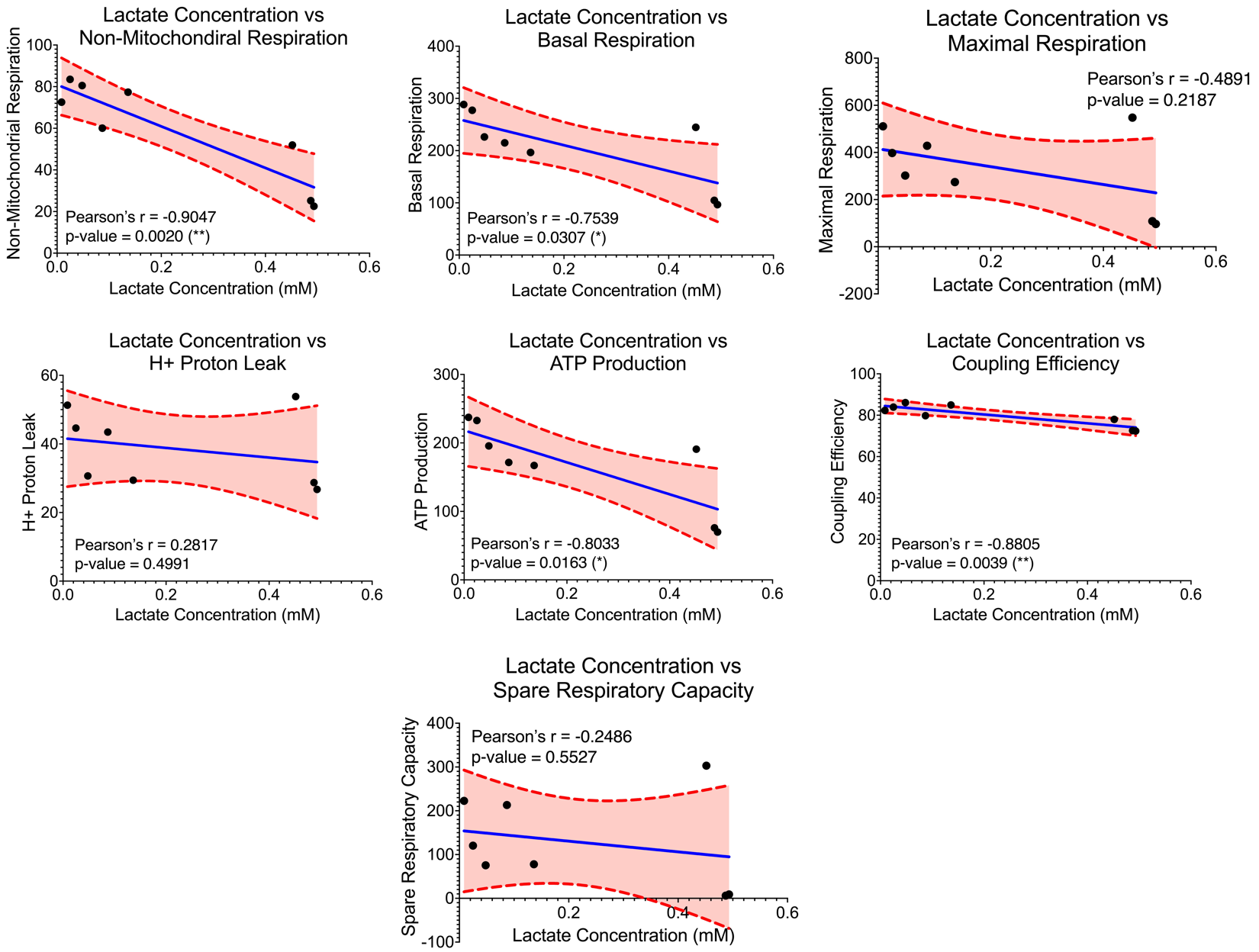


**Supplemental Figure 2. Correlations between lactate concentration and OCR parameters**. * p ≤ 0.05; ** p ≤ 0.01; Linear regression line (blue) with 95% confidence intervals (red). Graphs were made in GraphPad Prism®


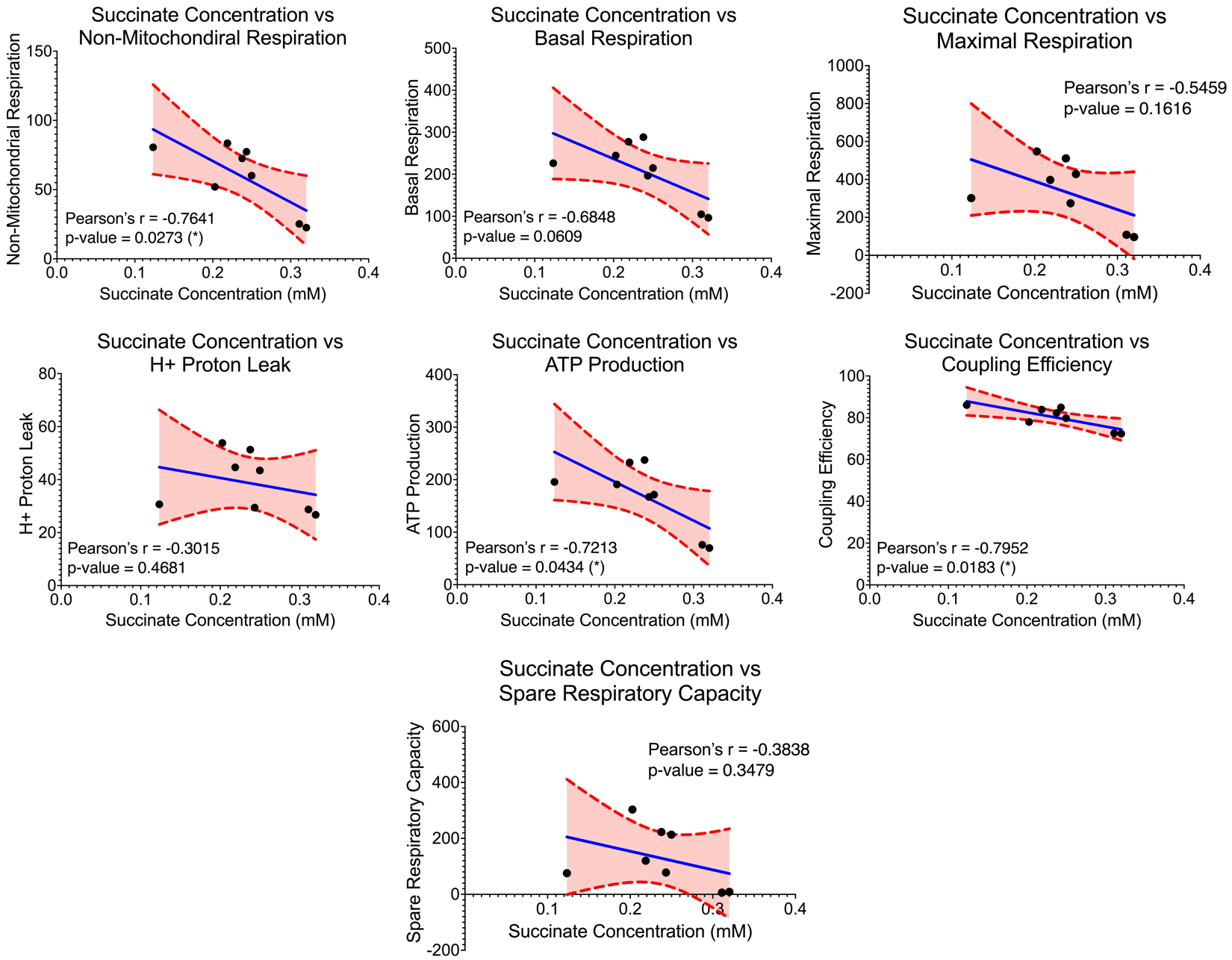


**Supplemental Figure 3. Correlations between succinate concentration and OCR parameters.** Linear regression line (blue) with 95% confidence intervals (red). Graphs were made in GraphPad Prism®.


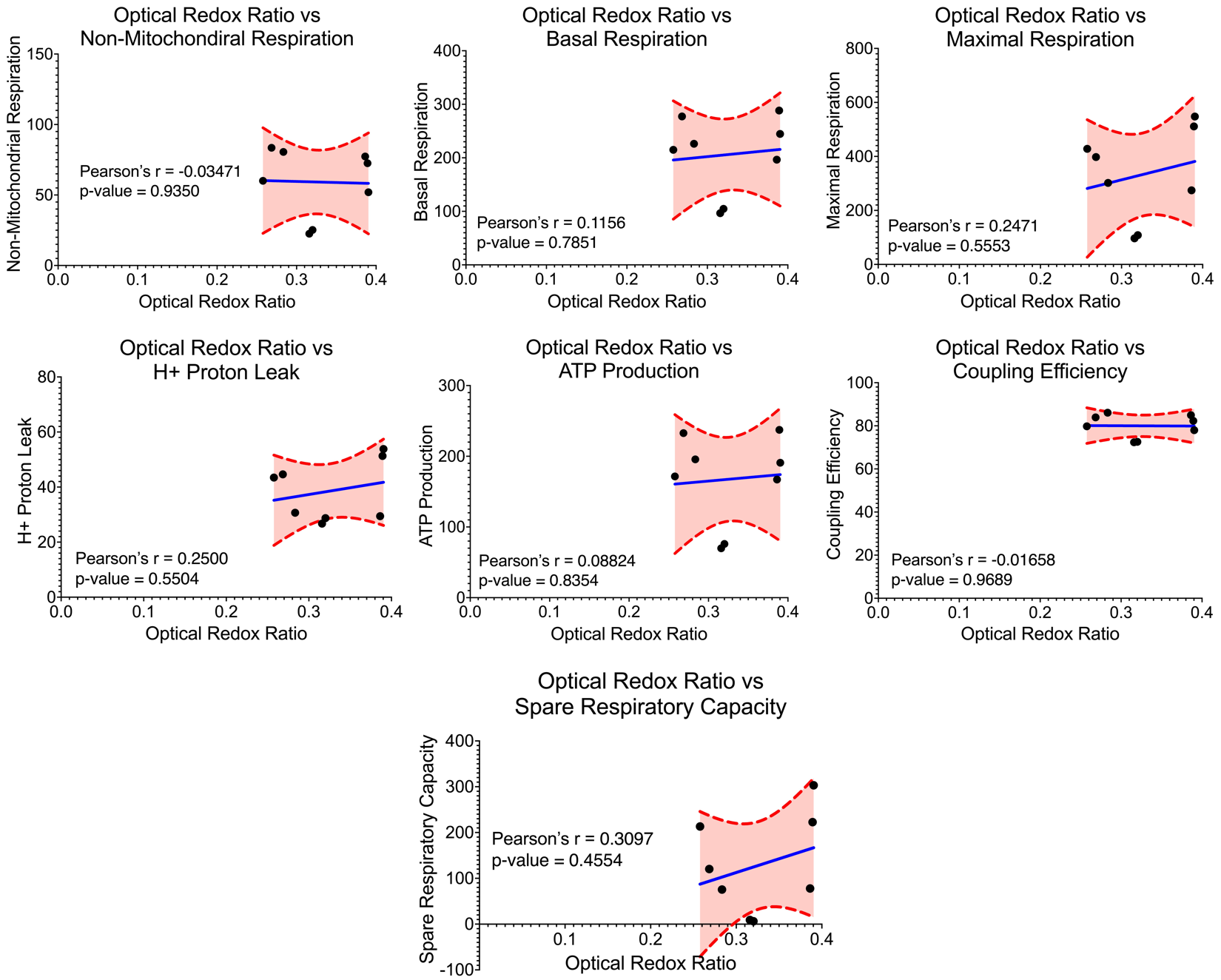


**Supplemental Figure 4. Correlations between optical redox ratio and OCR parameters.** Linear regression line (blue) with 95% confidence intervals (red). Graphs were made in GraphPad Prism®.


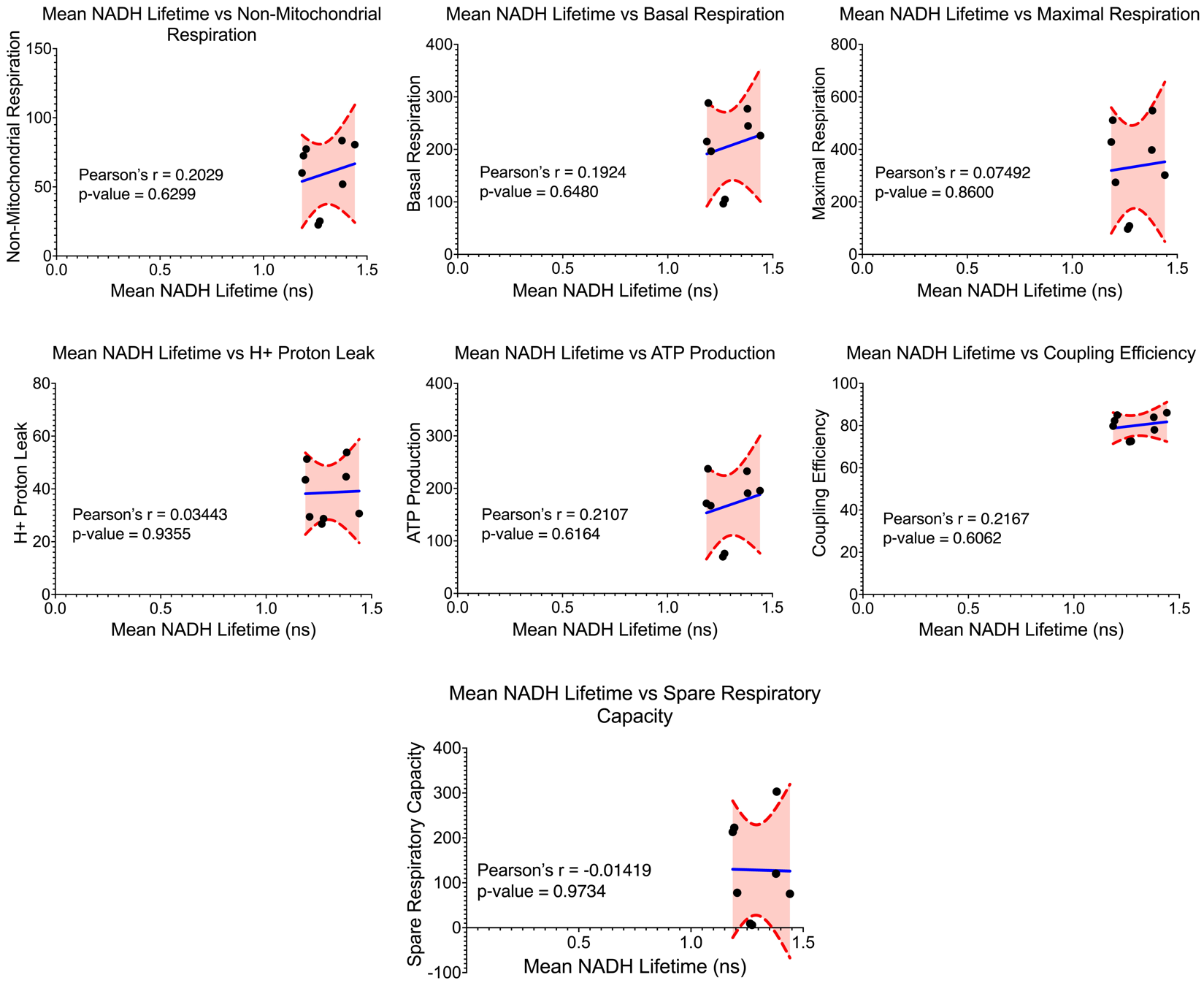


**Supplemental Figure 5. Correlations between mean NADH lifetime and OCR parameters.** Linear regression line (blue) with 95% confidence intervals (red). Graphs were made in GraphPad Prism®.


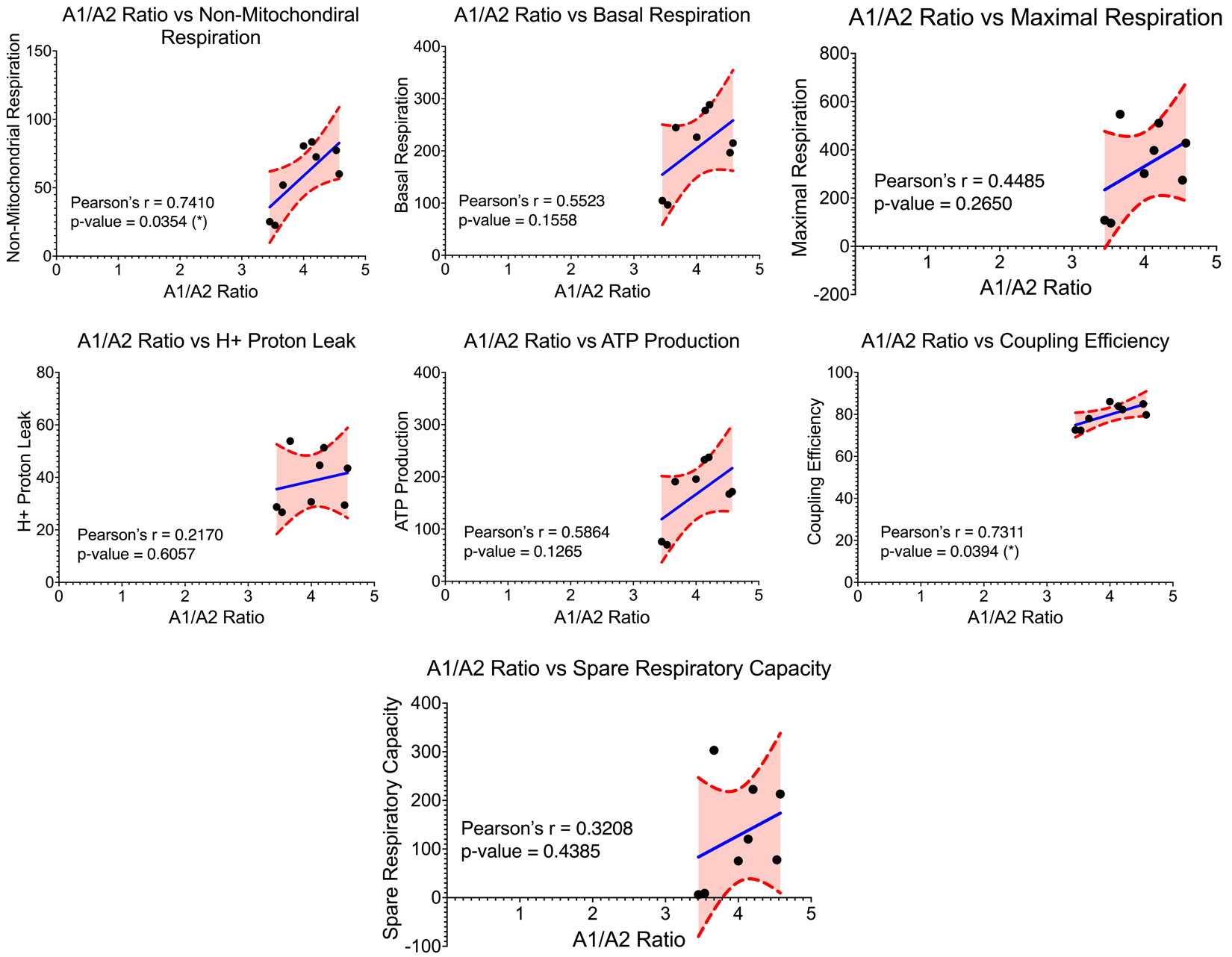


**Supplemental Figure 6. Correlations between A1/A2 ratio and OCR parameters.** * p ≤ 0.05; Linear regression line (blue) with 95% confidence intervals (red). Graphs were made in GraphPad Prism®


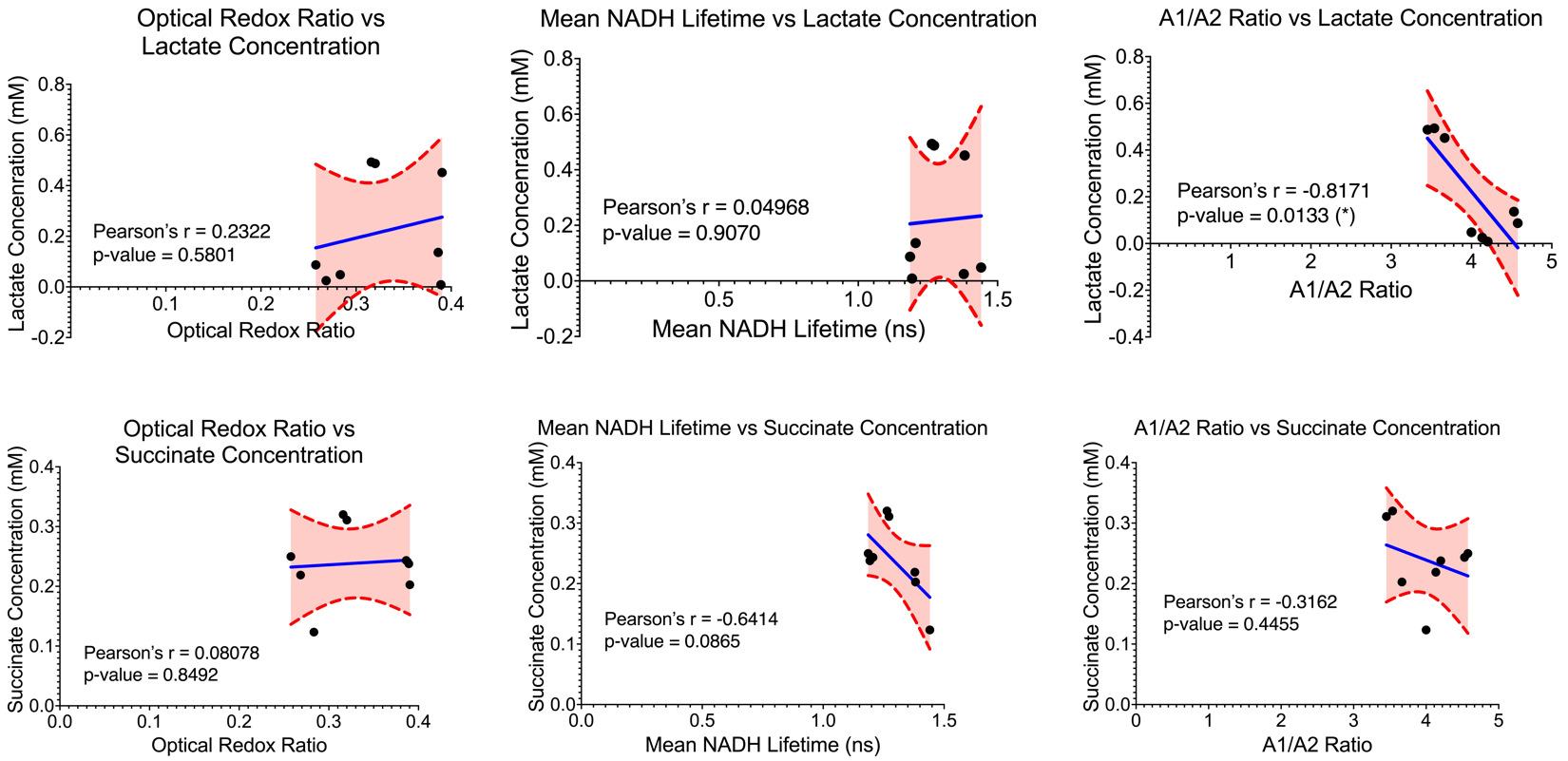


**Supplemental Figure 7.** **Correlations between lactate/succinate concentration and optical metrics (optical redox ratio, mean NADH lifetime and A1/A2 ratio).** * p ≤ 0.05; Linear regression line (blue) with 95% confidence intervals (red). Graphs were made in GraphPad Prism®
